# Multidimensional Latent Space Item Response Models: A Note on the Relativity of Conditional Dependence

**DOI:** 10.1017/psy.2025.5

**Published:** 2025-02-26

**Authors:** Inhan Kang, Minjeong Jeon

**Affiliations:** 1 Department of Psychology, College of Liberal Arts, Yonsei University, Seoul, Republic of Korea; 2 Social Research Methodology, Department of Education, School of Education and Information Studies, University of California, Los Angeles, CA, USA

**Keywords:** conditional dependence, item response theory, latent space model, multidimensionality, person–item interactions

## Abstract

Conditional dependence (CD) reflects potential interactions between persons and items in measurement, offering valuable information for deriving personalized diagnoses, evaluations, and feedback. The recent integration of psychometric models with latent space provides an effective way to visualize and quantify person–item interactions unexplained by latent variables and item parameters. In such applications, it is important to recognize the relative nature of CD, as models with different structures and complexities (e.g., due to factor dimensionality and item parameters) produce varying systematic explanations of person and item effects, leading to differing residual variations in both quantitative and qualitative sense. To demonstrate this relativity, we extend the previously developed unidimensional Rasch-based latent space item response model by incorporating between-item multidimensionality and item discrimination parameters. The resulting model can be reduced to simpler models with appropriate constraints, allowing us to explore the relativity in CD by comparing them. Simulation studies demonstrate that (1) the most complex proposed model properly recovers its parameters, (2) it outperforms the traditional IRT models by accounting for CD, and (3) the models in comparison exhibit distinctive extents of CD. The study continues with empirical examples that further illustrate relative changes in the extent and configurations of CD with practical implications.

## 1 Introduction

The violation of the conditional independence (CI) assumption, referred to as conditional dependence (CD), has become one of the primary research topics in the field of psychological and educational measurements. The CI assumption states that covariations between item responses can be systematically and fully explained through latent variables and item parameters. In contrast, CD suggests the presence of item response covariations beyond these explanations. Minor degrees of CD might be considered a form of randomness that cannot be explained by model parameters and thus ignored. In contrast, substantial CD could imply that relying solely on models with the CI assumption risks overlooking a considerable portion of the information inherent in the data. In the latter cases, it becomes necessary to seek alternative models or appropriately modify the current ones to analyze the information encapsulated under CD.

In particular, CD reflects data variations due to *person–item interactions* unexplained by person and item effects in a model. For a simple analogy to see this (Jeon et al., 2021), consider the standard Rasch model, 

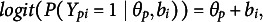

where 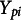 is a binary response of person *p* to item *i*, 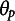 is a latent variable for person *p*, and 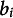 is an intercept for item *i*. This is consistent with a two-way analysis of variance (ANOVA) with the logit link function and two additive main effects, but no interaction. A latent variable 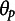 explains the main person effect (which does not vary across items) and an item parameter 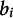 explains the main item effect (which does not vary across persons). CD, as a part of residual variations unexplained by 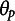 and 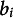 in the model, reflects some person–item interactions. These interactions indicate that item characteristics (e.g., difficulty, discrimination) may vary across individuals, and a person’s abilities or attributes may yield different effects depending on the item. Therefore, the information implied by CD can provide crucial insights for analyzing the detailed effects of specific persons and items.

Research in psychometrics has explored and analyzed CD from various perspectives. The approach of primary interest in this article is the latent space item response model (LSIRM; Jeon et al., 2021). This model integrates the standard Rasch model, used for analyzing psychometric data, with latent space models traditionally used for analyzing network data. The model assumes that persons and items can be mapped onto a shared metric space called a latent space (also known as an interaction map). The core function of this approach lies in exploring unexplained interactions between persons and items via their positions and distances on the latent space (while analyzing the main person and item effects with the Rasch model parameters), through which personalized information tailored to specific individuals and/or specific items can be extracted. Whereas traditional research topics related to CD, such as measurement invariance and differential item functioning, focus on group-level differences in item effects, the latent space approach offers greater potential in addressing individual-level differences in item effects and further allowing for the analysis of individual differences, individual profiles, and person–item interactions. Since the study by Jeon et al. (2021) that focused on binary responses, latent space models have been extended to encompass binary responses and response times (Kang et al., 2023), as well as non-binary responses (Kang and Jeon, under review).

### 1.1 The relative nature of CD

An important characteristic of CD that has not been richly discussed in the literature is its relativity: the extent of CD is relative to model complexity due to main model parameters, such as latent variables and item parameters in psychometric models. These main-effect parameters account for person and item effects, providing *systematic* ^1^ explanations for some parts of the total data variations.^2^ The remaining part of data variations (i.e., residual variations) would stem from other sources rather than the main effects already taken into account. These sources include CD as well as pure random noise. From this perspective, CD reflects a part of the data information left unexplained even after controlling for the current main effects.

Consequently, the extent of CD can vary depending on the parameters implemented in a model. This implies that certain aspects of CD can be systematically explained and removed by incorporating appropriate person or item parameters. For instance, adding item discrimination parameters to a standard Rasch model is expected to increase the part of data variations systematically explained by the model, thereby reducing the unexplained CD. Similarly, if a large part of CD arises from prior knowledge about items, employing parameters capturing these effects could transform this part of CD into systematically explained effects. Even some unexplained person–item interactions can be absorbed into systematic explanations if they can be captured by, for example, a product of the main person and item parameters. Furthermore, modifying the model may sufficiently reduce the extent of CD, making the resulting model satisfy the CI assumption. However, if there are person–item interactions that cannot be accounted for by main person and main item effects, these would remain unexplained. To summarize, the following decomposition of data variations by a psychometric model can be suggested: 
(1)

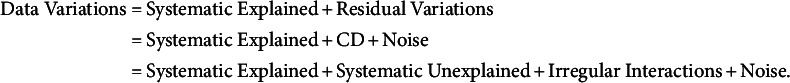



The relative nature of CD means that the components in Equation (1) can differ in their extents by the choice of main-effect components taken into consideration in a psychometric model. This is a critical consideration when aiming to analyze and utilize CD, because the choice can influence the information extracted regarding person–item interactions. If different models produce different extents of CD, personalized diagnoses and evaluations inferred from CD would differ accordingly. Furthermore, if a model has insufficient main effect parameters, it might be more appropriate to prioritize expanding systematic explanations of data variations through model modifications (i.e., adding appropriate main effect parameters) rather than adhering to the existing model and analyzing the unexplained CD based on it.

The decomposition of data variations by psychometric models described above is analogous to the well-known decomposition of the sum of squares in linear regression (with fixed effects) in which the total sum of squares (SST) is split into the regression sum of squares (SSR) and the error sum of squares (SSE). If the regression assumptions hold, this decomposition would suffice. However, if there are some violations, SSE would reflect not only pure random variations due to noise but also variations due to other sources. These include some “systematic” model violations such as non-zero shifted expectations of errors and heterogeneity in error variance due to, for instance, omission of necessary predictors and non-normal distribution. Also, although a basic regression model assumes the same regression coefficients for all respondents, the actual effects may exhibit irregular between-respondent differences implying respondent-variable interactions (which might be modeled with random effects under statistical assumptions). All these suggest the following conceptual decomposition, which corresponds to Equation (1) for measurement models: 
(2)

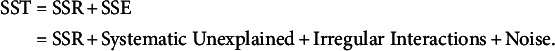



This analogy highlights an important point in studying CD: main effect parameters should be carefully selected and added to psychometric models to provide appropriate information regarding person effects, item effects, and person–item interactions implied by CD. As more predictor variables are added into a linear regression model, SSR rises and SSE falls, but blindly adding predictor variables can lead to overfitting and lack of interpretability. To psychometric models, this implies a trade-off in modeling CD: incorporating more person or item parameters into psychometric models can reduce CD but the additional parameters should be precisely estimated and provide meaningful insights into persons and items in a stable way. If not, an alternative way of looking into CD (e.g., via a latent space) could be a better option.

### 1.2 Extensions of the LSIRM

The relativity of CD discussed so far offers several implications for the application of the LSIRM. As the LSIRM is based on the standard unidimensional Rasch model, it has only a few main effect parameters, facing some restrictions in systematically explaining data variations. Consequently, there is a possibility that the latent space might detect excessively large CD some of which could be explained by carefully incorporated person and item parameters. In this regard, this article explores two potential extensions of the LSIRM and, based on these, discusses the relativity of CD.

The first extension involves the introduction of multidimensional latent variables. Many tests, exams, and measurement tools aim to simultaneously assess multiple psychological constructs to investigate their interrelationships. The overall performance of a respondent can be evaluated for each of these constructs. The existing LSIRM, however, employs a unidimensional latent variable that would, when applied to data involving multiple factors, only capture an average tendency of respondents across all factors. The strength of the LSIRM is that specific characteristics of unspecified factors may still emerge through the latent space even in this case, manifesting as clusters of items corresponding to each factor (Kang & Jeon, 2024). However, simply introducing multidimensional factors can produce a whole different result on a latent space by systematically explaining this type of CD due to unspecified factors. After this modification, the latent space would be able to capture item correlations beyond the multidimensional nature of the current measurement tool, facilitating a more detailed analysis of person–item interactions and individual characteristics. Therefore, when the factor dimensionality of the measurement data is known a priori, employing a multidimensional model can lead to a more accurate analysis of the extent of CD and the unexplained person–item interactions, as well as the main person and item effects.

The second extension involves incorporating item discrimination parameters, as in the two-parameter logistic IRT model (2PLM). This addition allows for analyzing a distinct item characteristic other than item difficulty and helps to partial out data variations attributable to the corresponding main item effect. More importantly, unlike item difficulty, item discrimination is directly related to the person–item interactions implied by CD. In IRT models, discrimination parameters are included as multiplicative factors with the latent variable, capturing aspects of person–item interactions that can be modeled as a product of person and item effects. In other words, by incorporating item discrimination parameters, the model can systematically account for some part of person–item interactions through the combination of person and item parameters. Consequently, this approach is expected to yield a different extent and configuration of CD, providing more refined information on unexplained person–item interactions compared to the standard Rasch model.

Based on these considerations, this article has a bi-fold aim: to propose the Multidimensional LSIRMs (MLSIRMs) and discuss the relative nature of CD. The MLSIRMs integrate multidimensional IRT models with a latent space. The proposed models are direct extensions of the LSIRM and the parameter spaces of their main model parameters are nested. By imposing appropriate parameter constraints on these models, it is possible to derive the LSIRM as well as several other variations of latent space models. Using this nested structure, we aim to compare CD and person–item interactions that arise from these different versions of latent space models to discuss the relative nature of CD.

This article is organized as follows. First, we introduce the MLSIRMs and their relevant estimation methods. Next, we conduct simulation studies to explore the statistical properties of the MLSIRMs, assess the impact of inadequately accounting for substantial CD, and discuss the relative nature of CD across different latent space models. Following this, we provide empirical examples using the most complex MLSIRM and its nested variations to further illustrate the relativity of CD and underscore the importance of appropriate modeling, with examples of individual difference analysis through latent space. Finally, the article concludes with a discussion of related issues.

## 2 Model

### 2.1 Multidimensional Latent Space Item Response Models

Throughout the manuscript, 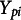 represents an item response of respondent *p* ( 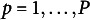 ) to item *i* ( 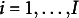 ). To derive extensions of the LSIRM, we start from the multidimensional IRT models with a logit link function (although a probit function or other choices can be used) such as the Rasch model and the two-parameter logistic model (2PLM), and integrate them with a latent space. First with the 2PLM, an extension of the LSIRM which we call the Multidimensional Latent Space 2PLM (MLS2PLM) can be expressed as follows: 
(3)

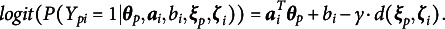



The logit of the response accuracy is modeled as a function of several parameters such as 1) 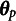 , a *D*-dimensional vector of latent abilities for person *p*, 2) 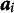 , a *D*-dimensional vector of discrimination parameters for item *i*, and 3) 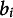 , an intercept for item *i* (usually interpreted as an overall easiness or an overall negative difficulty parameter for ability tests and as a symptom threshold parameter in clinical tests). These correspond to the multidimensional 2PLM. We restrict our interest to the cases where each item measures a single ability only, as in between-item multidimensional IRT models (Adams et al., 1997; Rijmen & De Boeck, 2005). Thus, the dimensionality concerned here corresponds to that in CFA models with a simple structure and no cross loadings. Accordingly, for each item, the vector 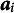 has only one non-zero element. For example, if item *i* measures the *d*th latent ability, then 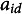 is non-zero and freely estimated whereas all other 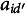 ( 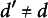 ) are set to zero. In this case, the first term on the right side of Equation (3) reduces to 
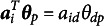
 . For simplicity, we sometimes denote 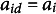 .

Another difference between the proposed MLS2PLM and the standard 2PLM is in the last term 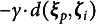 on the right side of Equation (3). The MLS2PLM assumes that persons and items can be mapped onto a shared *K*-dimensional metric space as in previous latent space modeling of psychometric data. Parameters 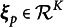 and 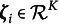 represent latent positions/coordinates of person *p* and item *i*, respectively. On the latent space, distances between persons, between items, and between persons and items can be computed. The function 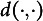 determines how these distances are computed. In this article, we use the Euclidean distance and set 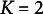 for visualization so that 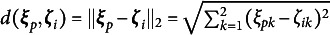 , as done in many previous latent space models (Handcock et al., 2007; Hoff et al., 2002; Jeon et al., 2021; Kang & Jeon, 2024; Kang et al., 2023; Smith et al., 2019). Distances are multiplied by the distance tuning parameter 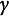 , which adjusts the scale differences between distances and linear predictor in the logit link function, and then included in Equation (3).

With the distance effect, the latent space can be utilized to extract valuable information to further understand the interactions between respondents and items as well as derive customized diagnoses and feedback for them. The distance effect is assumed to decrease logit accuracy. Thus, the larger the distance between person *p* and item *i* is, the more likely the person produces the incorrect response to the item. By visualizing the latent positions of persons and items in a single figure, one can obtain an interaction map with which person–item dynamics unexplained by latent abilities and item parameters can be studied. This map reveals that even respondents with similar levels of latent abilities can produce considerably different response profiles as the same item can have higher or lower probabilities of being solved by these respondents (Jeon et al., 2021; Kang et al., 2023). If items are from clinical scales, the interaction map can show that persons with similar overall clinical states might exhibit different symptom profiles as the same item can have higher or lower probabilities of being endorsed (Kang and Jeon, under review). Person–item distances can be computed for any person–item pair to formally quantify these kinds of observations. Then, more detailed diagnoses and treatments can be made for a specific person based on the distances from this person to the items.

Because distance has a negative relationship with response probability, we can instead define and rely on a similarity measure to ease straightforward interpretations of person–item interactions. To this end, we introduce a negative exponential decay function defined as follows: 
(4)

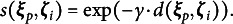



This transformed measure 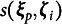 has a positive relationship with response probability. Specifically, a larger similarity indicates a smaller distance implying a relatively higher response probability, whereas a smaller similarity indicates a larger distance implying a relatively lower response probability. Also, Equation (4) maps positive-valued distances onto 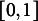 , producing scaled measures of person–item interactions that ease comparisons. It is worth noting that this transformation has been widely used in mathematical models in psychology, such as the SIMPLE (termed for scale-independent memory, perception, and learning; Brown et al., 2007) model of memory and Generalized Context Model (GCM; Nosofsky, 1986) of Categorization, as well as in clustering (e.g., Ng et al., 2001). Also, it can be derived from Equation (3) that 

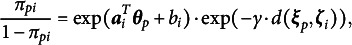

where 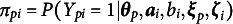 . This equation implies that the transformed measure in Equation 4 represents the decreases in the probability of 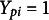 relative to that of 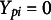 (i.e., odds) due to person–item interactions unexplained by the main model parameters.

It should be noted that the latent space in the proposed models is not the space of factors (latent variables or abilities). As Equation (3) shows, the model estimates factor scores 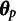 and latent positions 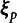 and 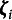 simultaneously. The factor score for respondent *p* provides the overall level of this respondent regarding the ability (or any psychological construct) being measured by items. Once estimated, this factor score does not depend on and vary across items, meaning that the factor score captures the global characteristics of the respondent. Beyond this information, the person–item distances 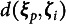 of the same respondent *p* to different items capture person–item interactions that vary across items. In this way, the distance effects account for item-specific variations and reactivities of the same respondent that are not captured by the factor score. These person–item interactions can be utilized to provide detailed information, diagnosis, and/or feedback for this respondent.

For more detailed descriptions of how to use the latent space models and their resulting interaction map for practical purposes, we refer the readers to some previous literature regarding the integration of latent space and psychometric models (e.g., Jeon et al., 2021; Kang & Jeon, 2024; Kang et al., 2023, see also Section 4.3. of the current article for examples of utilizing CD and latent positions). Below in this article, we focus on illustrating the nested models of the MLS2PLM, the relativity of CD with simulation-based studies, empirical applications, and theoretical discussions. All these compare estimated latent spaces from the MLS2PLM and its reduced models described in the following section.

### 2.2 Related models

Among the proposed MLSIRMs, the most complex MLS2PLM serves as a main framework for us to illustrate the relativity of CD because, with appropriate parameter constraints, it reduces to simpler models with larger expected CD. Primarily we focus on two constraints: (1) no item discrimination parameter, i.e., 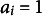 for all items ( 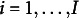 ) and (2) unidimensionality 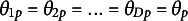 for all persons ( 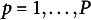 ).

Applying the first constraint, the MLS2PLM reduces to the multidimensional Rasch model with a latent space integrated. We will refer to this model as the Multidimensional Latent Space Rasch Model (MLSRM). Similarly applying the second constraint, the MLS2PLM reduces to the Unidimensional Latent Space 2PLM (ULS2PLM). Lastly, with both constraints applied, the MLS2PLM is simplified to the LSIRM (Jeon et al., 2021): 
(5)

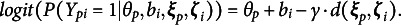

In this article, we will call this model the Unidimensional Latent Space Rasch Model (ULSRM), just for consistency with the other models. Table 1 provides a taxonomy of the model abbreviations for future reference.

**Table 1:** Taxonomy of the proposed multidimensional latent space item response models

	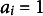 Fixed (Rasch)	Free 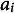 (2PLM)
Unidimensional: 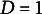	ULSRM	ULS2PLM
Multidimensional: 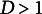	MLSRM	MLS2PLM

*Note*: The table provides the abbreviations of the model names. See the main text for the full names.

It would be worth noting that another constraint of 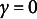 can reduce the models described above to traditional IRT models. Without latent spaces, these models assume CI and are not able to capture CD, beyond what can be explained by person and item parameters. Leaving CD unexplained may produce unwanted influences on the parameter estimation when CD is substantial. In this article, we demonstrate a potential bias in estimates due to CD by comparing the MLS2PLM, against its reduced version with 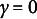 constraint and the CI assumption, which we will call the Multidimensional IRTl (MIRM) hereafter. Note that this is just the traditional multidimensional IRT model, and we focus on the multidimensional 2PLM in the following comparisons.

### 2.3 Inference

Most of the previous latent space modeling approaches have exploited Bayesian methods to estimate the model parameters. Also for the proposed models in this article, we developed a *Stan* (Stan Development Team, 2024) program, which utilizes the Hamiltonian Monte Carlo method for model estimation. The *Stan* code to fit the most complex MLS2PLM can be found in Section S1 of the Supplementary Material.

Samples from the joint posterior distribution can be obtained with the following specifications of the prior distributions as our recommendations: 
(6)

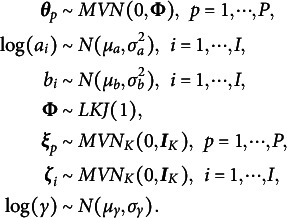



Here 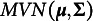 is a multivariate normal distribution with mean vector 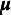 and covariance matrix 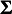 , with an appropriate dimension, 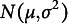 is a normal distribution with mean 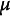 and standard deviation (SD) 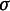 , and 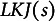 is a Lewandowski–Kurowicka–Joe distribution for a correlation matrix (Lewandowski et al., 2009) with a shape parameter *s*. To establish the identifiability of the model, the mean vector of the latent variables 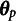 is fixed to the zero vector 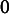 and means and SDs of the latent positions 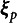 and 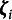 are set to 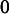 and 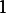 , respectively. Hyperparameters can be given appropriate hyperprior distributions or specific values. For item parameters, hyperpriors can be given as, e.g., 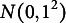 for 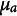 , 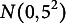 for 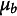 and 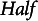 - 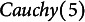 for 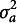 and 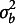 . Note that a prior on 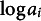 allows positive discrimination parameters only, implying that the probability of endorsing an item or giving the correct response increases as the latent trait/ability increases. This choice is based on the assumption that all items measure the target constructs appropriately (Baker, 1985) and reverse-keyed items are also reverse-coded before the main analysis. If a more general application is required, a normal prior can be imposed on 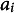 instead of 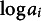 . For the log-transformed distance tuning parameter, 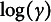 , 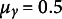 and 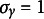 can be used as in the previous latent space approaches (Jeon et al., 2021; Kang et al., 2023), with which 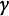 on its raw scale has mean of 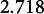 and SD of 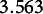 .

On the latent space, the Euclidean distance function 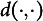 exhibits translational, rotational, and reflectional invariance with respect to the latent positions. Consequently, different configurations of these positions can yield identical distance values for all respondents and items. This issue can be resolved using matching methods commonly employed in multidimensional scaling. For the proposed models, we use Procrustes matching (e.g., Borg & Gorenen, 2005, Chapter 20). After obtaining posterior samples of all model parameters, we apply this method to each posterior sample of the latent positions. First, the posterior sample of model parameters with the highest log posterior density should be identified. The latent positions within this posterior sample can serve as the reference set. Then, configurations of latent positions in all other posterior samples can be aligned with this reference set while preserving the distances of all respondent-item pairs. Once the matching procedure is complete, the convergence of Bayesian chains can be assessed, and the posterior samples can be explored for further inferences. For practical applications of the Procrustes matching in the context of latent space modeling of psychometric data, we refer readers to the R package prolsirm, which is developed based on the R package MCMCpack (Martin et al., 2011).

### 2.4 Statistical test of CD using a slab-and-spike prior

If the main effect parameters such as latent variables and item parameters are insufficient to account for variations in item responses and the residual variations imply some person–item interactions, a latent space in the proposed models can account for some of these residual variations and yield useful information for diagnoses and evaluations. In contrast, in some cases, a model may already be equipped with sufficient main effect parameters to describe data variations, not requiring additional model-based mechanisms to capture CD (i.e., achieving CI). However, the distance effect assumed in the proposed models would always attempt to capture CD if a simple normal prior is given to γ as in Equation (6).

To address this concern, a regularization method with the slab-and-spike prior (Ishwaran & Rao, 2005; Mitchell & Beauchamp, 1988) can be given to the distance tuning parameter, as follows: 
(7)

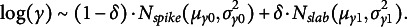



Following the previous applications of this prior to the latent space models (Jeon et al., 2021; Kang et al., 2023), we use 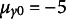 , 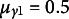 , and 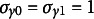 , which lead 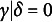 (spike) to have a distribution with mean of 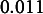 , mode of 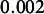 , and SD of 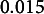 and 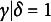 (slab) to have mean of 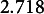 , mode of 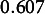 , and SD of 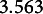 . Thus, if 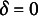 is selected, the prior effectively shrinks 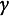 to zero, removing the distance effect on the logit accuracy from the model. In contrast, if 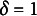 is selected, latent positions and distance effects can be well estimated without large shrinkage toward zero.

One complication in using the slab-and-spike prior with *Stan* is that the program does not support sampling of a discrete parameter like 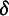 . As an alternative, 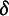 can be marginalized to produce a mixture distribution of 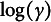 , with 
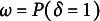
 and 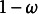 as choice proportions of the two components of the slab-and-spike prior. In this case, 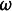 can be given a prior such as 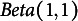 for estimation. Also, the posterior inclusion probability (PIP) of 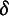 can be obtained as 
(8)

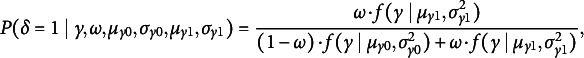

where 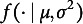 is a density function of 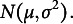


For latent space modeling of psychometric data, simulation-based studies have shown that the slab-and-spike prior can correctly detect significant CD and reject ignorable residual variations (Jeon et al., 2021; Kang and Jeon, under review; Kang et al., 2023). Thus, in this article, we employ this prior in our simulation studies and empirical illustrations of examining the relativity in CD.

### 2.5 Model complexity and relativity in CD

As discussed in the Introduction, psychometric models utilize latent variables and item parameters to account for person and item effects within the overall data variations. Also, by combining these main model parameters (e.g., the product of latent variables and item discrimination parameters), the models may capture some systematic person–item interactions. The remaining residual variations may imply CD and furthermore, person–item interactions that cannot be fully explained by simply combining the main effect parameters. These are the primary focus of the latent space approach.

According to this decomposition of data variations, model complexity plays a crucial role in balancing systematic and residual variations, providing some predictions for the MLSIRMs and their nested unidimensional models. Latent space models with fewer main effect parameters are more likely to detect larger CD due to reduced systematic explanations, whereas those with more main parameters would identify reduced CD or even reject it. Among the four models to compare, it is anticipated that the MLS2PLM will generally exhibit the smallest extent of CD, while the simplest ULSRM will show the largest extent. Comparing the MLSRM with the ULS2PLM has some complexity, as their main model parameters are not nested and can account for different types of main effects. Hence, the result of this comparison would be context-dependent and may vary by the appropriate dimensionality of the measurement data, item properties related to the slope of the item characteristic curve, etc.

However, this prediction is not without exceptions. Despite the similarity between the decomposition of 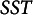 in linear regression and that of data variations by psychometric models, increases or decreases in model complexity of psychometric models do not always correspond to reductions or enlargements of residual variations and CD, due to several complications. This complexity arises because psychometric models often face greater uncertainty in parameter estimation compared to linear regression models. In contrast to 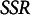 and 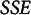 in linear regression, which have closed-form solutions and monotone relationships with the number of predictor variables, systematic and residual variations in psychometric models lack such simplicity and are linked to model complexity in much more intricate ways. Furthermore, there might be various sources of data variations that cannot be captured by specific person or item parameters. For the models in our examination, if there are unknown data variations that cannot be explained by adding multidimensional factors or item slope parameters, previous predictions about the extent of CD may no longer apply.

Note that the discussion above concerns possibilities rather than established findings. To explore and investigate these possibilities, we proceed with simulation studies and empirical examples using the proposed MLSIRMs and their simplified variations. These models will serve as our primary tools to examine the relative nature of CD.

## 3 Simulation studies

We conducted a series of simulation studies to (1) examine parameter recovery of the MLS2PLM under substantial CD, (2) illustrate influences of not accounting for non-negligible CD on parameter estimation, and finally, (3) demonstrate the discussed relativity of CD by comparing LS models with different model parameters and complexities.

### 3.1 Parameter recovery

#### 3.1.1 Data generation and analysis

For the first simulation study, we generated data from the MLS2PLM with the data-generating parameter values sampled or determined as follows. Item discrimination parameters 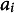 were given *I* numbers evenly dividing the interval 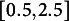 and item difficulty parameters 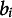 were similarly given *I* numbers evenly dividing the interval 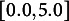 , but the numbers were randomly permuted. The latent ability scores 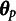 were sampled from a multivariate normal distribution with zero mean vector and covariance matrix 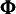 with 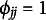 and 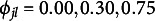 ( 
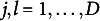
 , 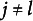 ). To examine the parameter recovery under CD, we used latent positions randomly sampled from the standard multivariate normal distribution and the distance tuning parameter of 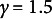 . We repeated data generation across simulation conditions with the number of persons 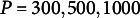 , the number of factors 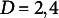 , and the number of items per factor 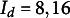 , which yielded 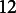 conditions. For each condition, we generated 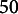 synthetic datasets for repetitions.

We fitted the MLS2PLM to each synthetic dataset with the inference method described in Section 2.3. We sampled from the joint posterior distribution of the MLS2PLM with three Bayesian chains, each with 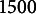 iterations. The first 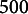 iterations from each chain were discarded for burn-in. To ensure convergence, we examined the potential scale reduction factor ( 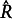 ; Gelman, 1996; Gelman et al., 2013) with 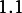 as its cutoff and visually inspected posterior densities, which did not reveal any convergence issue.

#### 3.1.2 Results

To assess parameter recovery, we obtained the point estimates of the model parameters from the posterior chains and computed their mean squared error (MSE), bias (evaluated with the absolute difference between estimates and true values), and standard error (SE). The calculation was done for each parameter but averaged across items (for 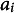 , 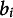 , 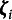 ), across persons (for 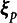 ), across persons and factors (for 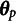 ) and across matrix elements (for 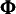 ) to summarize results. For latent positions, their values were also averaged across 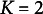 dimensions.

Table 2 presents the recovery results with 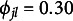 . The other recovery results (with 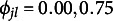 ) can be found in Section S2 of the Supplementary Material. The results show that the MLS2PLM can recover its parameters reasonably well. Statistics of person parameters ( 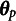 and 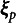 ) were a bit large when the number of items is small (e.g., 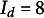 ), but these values were comparable to previously reported MSE values of unidimensional 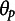 in the parameter recovery study of the 2PLM without CD (0.4–0.7; Hulin et al., 1982; Natesan et al., 2016; Stone, 1992). Also, recovery showed expected improvements as the number of items increased. Estimation of 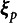 became more accurate with a larger *D* ( 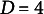 ), because the same latent positions can be constrained with more items ( 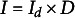 ). However, for 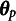 , a larger *D* did not improve the estimation because it introduced more factors to estimate. In fact, what was important for 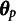 was the number of items per factor 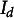 , not the number of total items *I*.

**Table 2:** Parameter recovery results of the multidimensional latent space two-parameter logistic model

			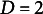							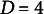						
Measure	*P*	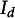	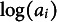	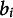	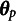	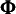	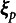	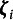	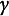	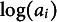	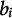	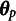	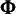	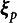	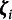	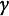
MSE	300	8	0.076	0.272	0.403	0.014	0.630	0.245	0.013	0.050	0.160	0.356	0.007	0.343	0.101	0.004
		16	0.048	0.182	0.270	0.003	0.341	0.100	0.003	0.042	0.201	0.252	0.007	0.209	0.103	0.001
	500	8	0.046	0.247	0.375	0.003	0.544	0.130	0.011	0.035	0.134	0.385	0.003	0.345	0.086	0.002
		16	0.030	0.107	0.263	0.002	0.349	0.066	0.002	0.022	0.099	0.235	0.002	0.183	0.051	0.002
	1000	8	0.023	0.105	0.390	0.004	0.502	0.053	0.006	0.020	0.081	0.373	0.002	0.334	0.040	0.002
		16	0.017	0.086	0.244	0.001	0.348	0.041	0.002	0.013	0.063	0.232	0.005	0.179	0.028	0.000
Bias	300	8	0.124	0.308	0.364	0.091	0.509	0.223	0.017	0.102	0.189	0.319	0.047	0.283	0.135	0.007
		16	0.103	0.243	0.274	0.032	0.271	0.111	0.010	0.092	0.200	0.234	0.067	0.174	0.096	0.005
	500	8	0.086	0.311	0.344	0.008	0.409	0.154	0.014	0.067	0.171	0.346	0.023	0.277	0.116	0.019
		16	0.063	0.102	0.262	0.020	0.266	0.070	0.012	0.047	0.125	0.215	0.019	0.148	0.072	0.002
	1000	8	0.036	0.154	0.357	0.048	0.388	0.065	0.024	0.045	0.134	0.325	0.024	0.267	0.076	0.021
		16	0.048	0.121	0.240	0.006	0.282	0.063	0.012	0.037	0.090	0.203	0.060	0.149	0.043	0.004
SE	300	8	0.220	0.358	0.407	0.072	0.431	0.327	0.112	0.187	0.294	0.417	0.063	0.430	0.252	0.063
		16	0.169	0.307	0.362	0.045	0.435	0.262	0.055	0.160	0.305	0.382	0.045	0.371	0.242	0.036
	500	8	0.186	0.311	0.398	0.049	0.462	0.266	0.102	0.163	0.266	0.417	0.050	0.439	0.221	0.043
		16	0.142	0.274	0.367	0.033	0.443	0.230	0.048	0.133	0.256	0.379	0.033	0.357	0.196	0.040
	1000	8	0.140	0.275	0.404	0.042	0.463	0.212	0.074	0.124	0.227	0.427	0.035	0.432	0.165	0.040
		16	0.110	0.219	0.374	0.022	0.442	0.164	0.040	0.103	0.202	0.381	0.024	0.352	0.145	0.021

The values of MSE, bias, and SE for the other parameters were reasonable and exhibited the anticipated effects due to the simulation conditions. For example, item parameters were generally estimated more accurately with a larger number of persons, and person parameters improved with more items. Notably, although having more items means more item parameters to be estimated, all item parameters ( 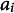 , 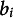 , and 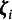 ) were estimated more accurately with larger 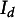 and *I*. This would be because the additional items provided better constraints on the person parameters, which in turn, improved the calibration of item parameters.

It is important to note that this simulation study examined parameter recovery under the effect of CD. The results show that the main model parameters to capture person and item effects are not much affected by CD when a model with a latent space is employed to account for unexplained data variations implied by CD. If a model is not equipped with a component to capture CD, its effect can propagate to the recovery of the main model parameters in an unwanted way. In the next simulation study, we perform similar parameter recovery but compare the MLS2PLM and the MIRM to illustrate this point and further demonstrate the advantages of incorporating a latent space in modeling measurement data.

### 3.2 Impact of ignoring CD

In the second simulation study, we aimed to demonstrate the advantages of implementing a latent space for parameter recovery. To this end, we fitted the MIRM, which has the same main parameters as the MLS2PLM but does not employ a latent space, to the synthetic datasets used in the first simulation study. To balance computational efficiency with the main objectives of the study, we chose four conditions with 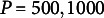 , 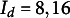 , and 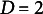 (i.e., the total number of items were 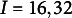 ). The MIRM was fitted to the synthetic datasets using the same Bayesian estimation method as the MLS2PLM except that latent positions and distance tuning parameters were excluded. Consequently, the MIRM could not account for any residual dependencies, particularly person–item interactions that cannot be explained by the product of item discrimination parameters and latent abilities. The key question we sought to address was how much the person and item parameters of MIRM would be influenced by not accounting for the distance effect and CD considered in the data-generation process.

**Figure 1:**
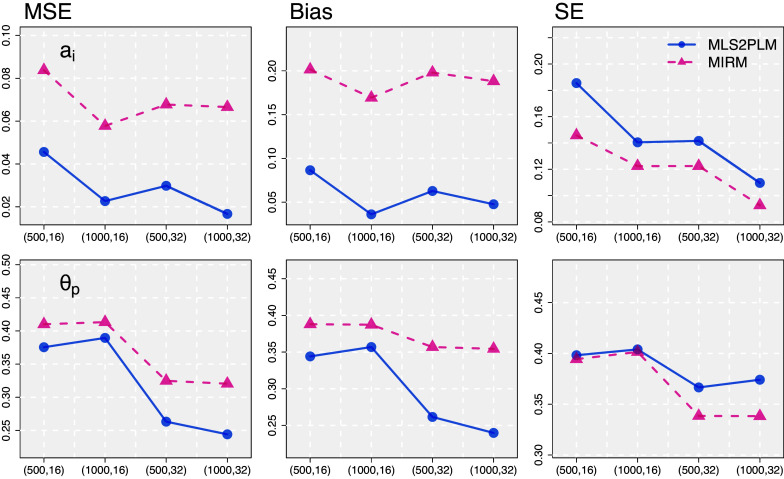
Comparison of parameter estimation.

We compared the MLS2PLM and the MIRM based on MSE, Bias, and SE of the estimates. Figure 1 shows the results. The left, middle, and right panels present results for MSE, Bias, and SE, respectively, as denoted on top of the upper panels. The upper and lower panels present results for 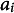 and 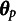 , respectively, as denoted at the top-left side of the left-most panels. In each panel, the blue circles with solid connecting lines and the red triangles with dashed connecting lines represent results from the MLS2PLM and the MIRM, respectively. Each panel has four points for each model, representing the computed values of the measure across four conditions as shown as 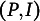 on the *x*-axis.

As shown in the figure, we restricted the comparison to the two parameters 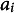 and 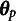 and excluded 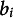 . This was because 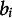 estimates from the MIRM are not comparable to the data-generating 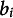 values used for the MLS2PLM. Due to the negative distance effect, 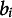 of the MLS2PLM is typically much larger than those in the MIRM, unless the CD is very small. Simply put, overall sizes of 
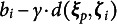
 correspond to those of 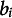 in the MIRM, but the former vary across persons, making comparisons with the latter and evaluations of bias infeasible.

In general, the results show that the MLS2PLM outperformed the MIRM. Estimates from the MLS2PLM had larger SEs due to the need to estimate more parameters and higher model complexity. However, they exhibited greater reductions in bias, resulting in considerable reductions in MSEs. Importantly, the impact of CD did not decrease with increasing data size; instead, it turned out from the simulation study that CD exerted larger influences on the MIRM estimates as the data size grew.

The results in Figure 1 can be investigated from an extended perspective based on model predictions. This is because the influences of CD on parameter estimates can be propagated to predictions generated by the models. To demonstrate that the proposed MLS2PLM can yield better predictive accuracy, we performed posterior predictive checking (PPC) with the MLS2PLM and the MIRM, computed predicted item-wise and person-wise response proportions, and examined which model produced better predictions by comparing predictions against data-based item-wise and person-wise proportions. To save space, we present the results in our Supplementary Material (see Section S3), which show that capturing CD with the integrated latent space approach can improve model predictions as well.

The comparison presented here might seem unfair given that the MLS2PLM was used as the true data-generating model. However, the MLS2PLM can still perform better than the MIRM as long as data imply any form of CD. This is because this latent space model can at least partially account for CD, whereas the traditional model cannot. Moreover, even if the data-generating process adheres to the CI assumption and implies no CD at all, the MLS2PLM can reduce to the MIRM with the slab-and-spike prior^3^.

Overall, the results have a clear implication that ignoring CD can distort parameter estimation in psychometric models to a great extent, potentially leading to incorrect inferences and conclusions. Using an integrated psychometric model with a latent space can facilitate more robust parameter estimation.

### 3.3 Relativity in the extent of CD

After establishing the appropriate parameter recovery of the MLS2PLM and demonstrating the utility of implementing a latent space in the context of parameter estimation, we proceeded to the third simulation study to illustrate the relative nature of CD. We again used the synthetic datasets generated in the first simulation study but limited our analysis to the four conditions used in the second study. The previously obtained results of the MLS2PLM were compared to the results from two reduced models, the ULS2PLM and the MLSRM, both fitted to the same datasets. Comparing the MLS2PLM to the ULS2PLM allows us to investigate the effect of underspecifing the number of factors. Similarly, comparison against the MLSRM reveals the impact of dropping the item discrimination parameters (i.e., fixing all 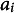 ’s to 1). The difference between the models may yield not only changes in the extent of CD but also substantive and qualitative differences in unexplained interactions (e.g., patterns of latent positions). However, for this simulation, we focus on the effect of the main model parameters on the extent of CD only, because the patterns of interactions can vary across repetitions, making it hard to consistently compare them for all three models and simulation repetitions.

Both competing models would not account for certain sources of person–item interactions, potentially producing increases in the extent of CD. To compare these effects quantitatively, we examined the 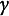 estimates ( 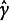 ) from the three models. Generally, we anticipate that models with constraints on latent variables and main item parameters would exhibit larger CD because they lose some of their capacity to systematically explain data variations. Consequently, overestimated 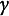 values are expected in most cases. However, exceptions may occur where 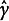 is larger in a more complex model due to randomness in estimation. This outcome contrasts with what is typically expected (and can be mathematically proven) in linear regression analysis.

Comparison of the models solely based on 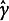 would need justification. The parameter 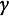 is simply a tuning parameter to adjust the scale difference between the Euclidean distance and the other model parameters. Typically, comparing 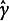 could not be meaningful in practical cases because its value can vary due to factors other than the extent of CD, such as differences in the underlying structure of latent positions, link functions, etc. For example, if one model detects largely deviant clusters of items or persons while the other model yields randomly distributed latent positions, it could be argued that the former model exhibits much larger CD even if its 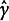 is smaller. In such cases, sizes of 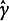 do not correctly represent the extent of CD. However, in the current simulation, data-generating values of latent positions were randomly generated from the standard multivariate normal distribution. Also, all models in comparison use the same logit link function. These constraints in simulation design help observe and evaluate changes in the extent of CD based on 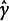 across three latent space models.

**Figure 2:**
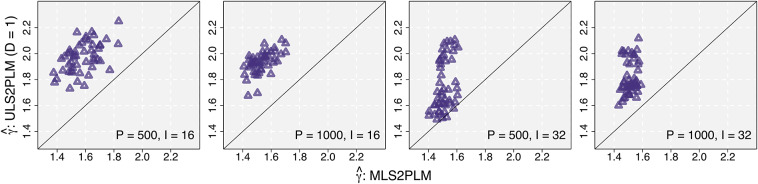
Comparison of estimated distance tuning parameters: MLS2PLM vs ULS2PLM, i.e., the effect of underspecifying the number of factors.

**Figure 3:**
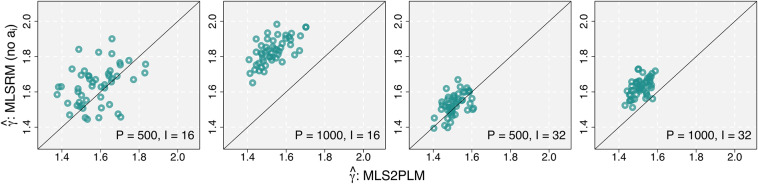
Comparison of estimated distance tuning parameters: MLS2PLM vs MLSRM, i.e., the effect of dropping the item discrimination parameters.

The comparison results are presented in Figures 2 and 3. In each figure, there are four panels of scatter plots corresponding to four conditions of *P* and *I*, as shown at the bottom-right side of each panel. In each panel, 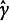 values from the MLS2PLM are plotted on the *x*-axis against those from the two competing models on the *y*-axis. The purple triangles in Figure 2 and the green circles in Figure 3 represent comparisons against the ULS2PLM and the MLSRM, respectively, as denoted by the *y*-axis labels. The diagonal line in each panel indicates the points at which 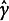 values from the MLS2PLM and the other competing models are equivalent. Hence, circles and triangles distributed on the top-left side of the diagonal line indicate that the simpler competing models detect larger CD and produce larger 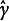 than the MLS2PLM. If this is the case, increases in CD can be attributed to the parameter constraints imposed on the simpler models and their resulting decreases in systematic explanations of data variations.

The results correspond to the anticipated relativity in CD as described in the introduction. The estimates 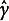 from the MLS2PLM were generally distributed around its data-generating value 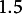 and as the data size increased, its estimation precision improved. The estimates from the competing models were mostly larger than those from the MLS2PLM. Specifically, the ULS2PLM that underspecified the number of factors produced larger CD than the MLS2PLM in almost all cases, with only two exceptions when 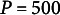 and 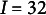 .

The MLSRM, which constrains all item discriminations to be 1, yielded a similar effect of increasing CD. However, there were some exceptions. Specifically, when 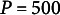 , the MLSRM produced smaller 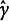 values than the MLS2PLM in 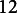 and 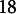 repetitions (out of 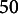 ) in the conditions with 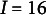 and 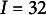 , respectively. When 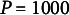 , overestimated 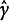 values from the MLSRM were more consistently observed, with only one exception in the condition with 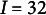 . Also, across both conditions of *P*, the MLSRM estimates of 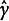 tended to be closer to those from the MLS2PLM when 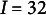 than when 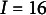 (i.e., the green circles were closer to the diagonal lines when 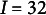 ). These findings align with expectations, considering the estimation issue with the item discrimination parameters. With larger numbers of persons (*P*), the MLS2PLM can more precisely estimate 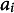 and detect the extent of CD. Consequently, there were fewer cases in which the MLS2PLM yielded larger error in estimating 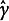 and the MLSRM produced smaller 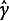 values. As the number of items (*I*) increases, more item discrimination parameters need to be estimated, which can lead to less precise estimates of 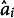 as well as less precisely estimated extents of CD. This could be associated with the observation that the MLSRM estimated smaller 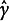 values than the MLS2PLM in more repetitions when 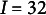 , compared to when 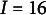 .

It might be tempting to compare the two results and examine the differences between the two constraints on CD. However, we do not pursue this here, as the actual differences can vary considerably depending on the number of underlying dimensions, the degree of misspecification, the distribution of true item discriminations, etc. A more thorough and comprehensive simulation study would be required to address this, which is beyond the scope of the current article.

## 4 Empirical illustrations

We continue to illustrate the relativity in CD as well as the practical utility of the proposed models, now with empirical examples. To this end, we utilized two datasets. The first dataset was from the Inductive Reasoning Developmental Test (IRDT; Golino & Epskamp, 2017). The IRDT dataset was collected from 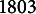 test-takers and used to illustrate an exploratory graph analysis (EGA) as a new way of estimating the number of latent dimensions. The IRDT has 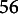 items to measure seven sequential stages of the development of inductive reasoning. Each stage was measured with eight items and item responses could be represented by a seven-factor structure. With this dataset, Golino & Epskamp (2017) showed that the EGA can be a better alternative to detect the number of factors than the traditional approaches used in exploratory factor analysis (EFA).

It is worth mentioning that the IDRT dataset was recently analyzed with the LSIRM (also denoted as ULSRM in the current article) to illustrate the utility of the model (Kang & Jeon, 2024). Noting that unspecified factors can be a data-based source of CD, it was shown that misspecified factors can emerge as item clusters in a latent space. Thus, a latent space can serve as another statistical tool to explore the dimension of factors. They also illustrated how to derive personalized diagnoses and evaluations for different respondents with similar latent abilities from the same data application.

The second dataset was collected from patients with Attention-Deficit/Hyperactivity Disorder (ADHD; Silk et al., 2019). From the Diagnostic Statistical Manual (DSM-5), the list of 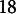 symptoms (nine inattentive and nine hyperactive) was obtained and the presence/absence of each symptom was measured from 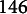 ADHD patients and 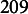 control subjects. A network approach implemented in the R package qgraph was applied to this dataset to explore the symptom network.

In our empirical illustrations, we applied the four latent space models (ULSRM, ULS2PLM, MLSRM, and MLS2PLM) to the two datasets. For the IRDT dataset, the multidimensional models (MLSRM and MLS2PLM) accounted for the factor structure with 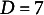 factors while examining CD with their latent spaces. Golino & Epskamp (2017) also analyzed this dataset using a seven-factor CFA model and a bi-factor model with seven specific factors to illustrate their similarities and dissimilarities from EGA. Similarly, as the ADHD dataset was concerned with inattentive and hyperactive symptoms, the multidimensional models examined this dataset with 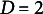 factors. The unidimensional models (ULSRM and ULS2PLM) employed a single factor for both datasets.

As the models differ in the number of main person and item parameters, they differ in their capabilities to systematically explain data variations, producing different extents of unexplained variations and configurations of latent spaces. This perspective has already been illustrated in the third simulation study. Now we further demonstrate it with real-world datasets, focusing on presenting and interpreting the estimated latent spaces.

In doing so, we first focus on the changes in the extent of CD in Sections 4.1 (IRDT) and 4.2 (ADHD). The resulting latent spaces exhibit differences not only in the extent of CD but also in the patterns of latent positions. This implies that, depending on which model is employed, utilizing CD via latent space can lead us to qualitatively different diagnoses for persons and items. These differences are examined and illustrated separately in Section 4.3.

For both data examples, we fitted the models with the Bayesian approach described in Section 2.3. We ran three Bayesian chains with 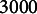 iterations, the first half of which were discarded for burn-in. Convergence was examined with 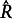 and visual inspection of posterior densities. The MLS2PLM with appropriate numbers of factors did not yield any convergence issues (see Section S5 of the Supplementary Material). The models with less flexibility produced rather large 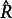 values in our initial attempt and sometimes failed to reach the convergence criterion. We resolved this by simply re-fitting the models with different initial values and obtained the results with convergence for all models.

### 4.1 Example 1: IRDT dataset

**Figure 4:**
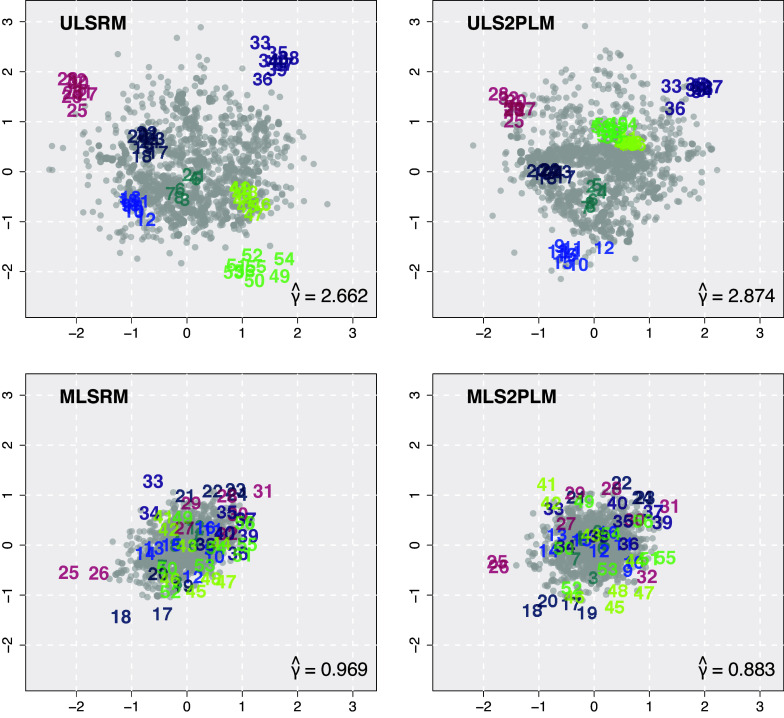
Estimated latent spaces for the IRDT dataset.

For the IRDT dataset, all four models selected the slab part of the slab-and-spike prior with the PIP 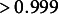 , implying substantial CD. Figure 4 shows the four latent spaces from four different models, as indicated at the top-left side of the panels. All panels have the same ranges for the *x*- and *y*-axes for comparison across the models. In each panel, the gray dots represent respondents and the colored numbers represent items. Items measuring the same factor (e.g., items 1–8) were given the same color code (e.g., dark cyan).

The results from the ULSRM (top-left) showed seven clusters of items, corresponding to the presumed seven-factor structure. As the model had a unidimensional latent ability that was supposed to influence all items, the differences between items measuring different factors remained unexplained by the main model parameters. These residuals were captured as the item clusters on the latent space, which corresponded to the specific factors used in the previous application of the bi-factor model (Golino & Epskamp, 2017). Some item clusters were close to each other, reflecting high correlations between their items beyond what can be explained by the common latent ability. Putting it all together, the latent space showed that the primary source of CD detected by the model would be the item clusters (i.e., unspecified factors). This finding generally replicated the result presented in Kang & Jeon (2024). When item discrimination parameters were incorporated (top-right), some item clusters merged, for instance, items 41–48 (lime) and 49–56 (green). This implies that some inter-cluster item correlations can be largely accounted for by item discrimination parameters, leading the corresponding clusters to seemingly merge. However, this did not entirely remove the clustering patterns of items.

The latent spaces produced by the multidimensional models exhibited an important distinction. Both MLSRM and MLS2PLM chose the slab part of the slab-and-spike prior, even after controlling for the effect of the seven underlying factors. In other words, the models detected substantial CD unaccounted for by correlations between those factors, and thus, the primary source of CD from the multidimensional models is not the underlying factors and their corresponding person effects. Due to this difference, configurations of the resulting latent spaces considerably changed. Most importantly, all items were intermixed regardless of which factor they were supposed to measure. This pattern was also consistent with the interpretation that variations due to the seven developmental stages of IRDT were not the main source of CD. From simple visual inspection, it seemed that incorporating item discrimination parameters or not did not yield noticeable differences in estimated latent spaces. Their differences may be revealed by more thorough investigations with quantification of interactions based on distances. An example of this with inter-item distances is provided in our Supplementary Material (Section S6.2).

#### 4.1.1 Reduction in the extent of CD: IRDT

The estimated value of the distance tuning parameter was 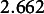 in the simplest ULSRM and changed to 2.877 (ULS2PLM), 0.969 (MLSRM), and 0.883 (MLS2PLM). The estimates did not always decrease as the model complexity with regard to the main effect parameters increased, but except for the comparison between ULSRM and ULS2PLM, the pattern was consistent with our anticipation and the simulation results. Notably, when the number of factors and the factor structure were correctly specified in the multidimensional model, the estimates were reduced to a large degree.

The spread of latent person and item positions showed similar reductions. For instance, the estimated positions from the unidimensional models are less spread than those from the multidimensional models in Figure 4. The left section of Table 3 presents the 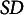 s of the estimated latent positions, quantifying this pattern in the spread. In general, 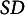 decreased as the model complexity increased, except that the MLS2PLM yielded slightly larger 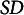 s than the MLSRM. Potentially, this could be compensated by the reduction in 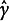 .

**Table 3:** Statistics related to Latent Positions. Left section: IRDT, right section: ADHD

	IRDT						ADHD					
Model	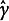	CI	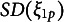	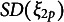	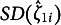	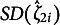	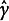	CI	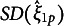	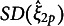	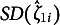	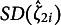
ULSRM	2.662	[2.535, 2.800]	0.797	0.792	1.255	1.346	1.493	[1.270, 1.745]	0.869	0.612	1.116	0.892
ULS2PLM	2.877	[2.674, 3.089]	0.744	0.750	1.003	1.061	1.569	[1.325, 1.832]	0.834	0.606	1.030	0.753
MLSRM	0.969	[0.813, 1.139]	0.372	0.303	0.692	0.636	1.345	[1.103, 1.601]	0.723	0.639	0.940	0.979
MLS2PLM	0.883	[0.719, 1.055]	0.364	0.315	0.712	0.695	1.415	[1.125, 1.729]	0.637	0.516	0.842	0.704

*Note*: CI: 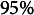 Credible intervals of 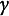 . SD: Standard deviations of estimated latent positions for persons ( 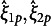 ) and items ( 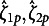 ).

**Figure 5:**
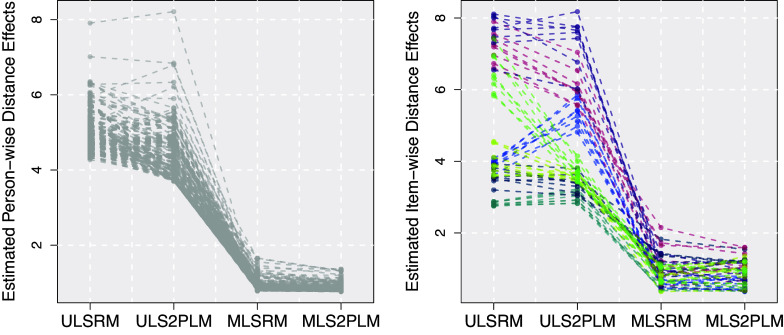
Reduction in the estimated distance effects 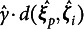 as a function of model complexity: The IRDT dataset. Left: Person-wise average distance effects. Right: Item-wise average distance effects.

Motivated by the findings described above, we took a deeper look into the reduction in CD based on the estimated interaction terms 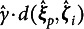 . We first computed all person–item distances, then averaged them first over persons and subsequently over items, resulting in person-wise and item-wise distance effects. This process was repeated for each model, providing each person and item with four distance estimates corresponding to the four models under examination. Figure 5 illustrates the changes in the distance effects, with person-wise estimates on the left panel and item-wise estimates on the right panel. In each panel, the *x*-axis lists the four models in the order of their model complexity, and the *y*-axis represents the averaged distance effects. Each dashed line corresponds to a single person or a single item. We used the same color codes as the latent spaces in Figure 4. Additionally, for clearer visualization, we randomly selected 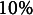 (approximately 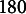 ) of the total sample for the person-wise estimates.

For both persons and items, the distance effects generally exhibited decreasing trends as a function of the number of person and item parameters incorporated into the model. The differences stemming from item discrimination parameters were weak and somewhat inconsistent, but the differences related to the dimensionality of factors were salient. Also, when the number of factors and the factor structure were adequately specified, the impact of item discrimination parameters became more consistent despite its small size. Overall, this result aligns with our expectations regarding the relativity of CD.

### 4.2 Example 2: ADHD dataset

**Figure 6:**
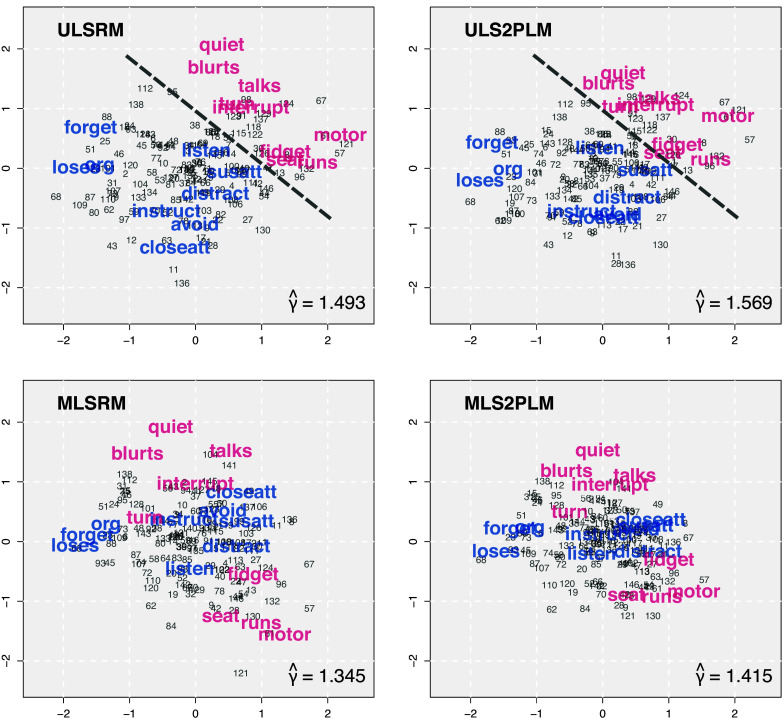
Estimated latent spaces for the ADHD dataset.

We analyzed the ADHD dataset as similarly as we did for the IRDT dataset to examine the relativity in CD. The primary goal was to replicate the findings from the IRDT dataset. Figure 6 shows the four latent spaces from the four models fitted to the ADHD dataset. The associated inter-item distance matrices are provided in the Supplementary Material (Section S6.2). All models yielded that the extent of CD was substantial with the PIPs 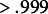 . In each panel, the gray numbers indicate respondents and words represent abbreviated symptoms used in the measurement. Section S6.1 of the Supplementary Material gives the full list of symptoms with their abbreviations, which can also be accessed from Silk et al. (2019, p. 4).

The figure suggests implications similar to those from Figure 4. The estimated latent spaces from the unidimensional models in the top panels showed greater variations across both persons and items. Also, items could be separated into two groups, e.g., by the dark gray dashed diagonal line added for reference. Unlike the results from the IRDT dataset, the clustering of items measuring the same factor was less distinct. This difference might be due to the ADHD dataset containing only two factors, compared to seven in the IRDT dataset. The two ADHD factors would generate relatively weaker data variations, making themselves less prominent sources of CD. Nevertheless, the two groups of symptoms remain distinguishable.

In contrast, the items in the latent spaces from the multidimensional models did not exhibit the same distinction. Specifically, the items measuring the hyperactive symptoms (red) were split into two groups, one distributed above the items for the inattentive symptoms and the other distributed below. This pattern was commonly observed in both multidimensional models. However, when item discrimination parameters were added, persons and items gathered more closely with each other.

#### 4.2.1 Reduction in the extent of CD: ADHD

As with the IRDT dataset, we performed further analyses to quantify the findings from the estimated latent spaces and look into the details of the reduction in CD. To this end, the estimated distance tuning parameter 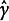 and 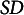 s of the estimated latent positions were calculated and presented in the right section of Table 3. The estimates for 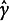 decreased when the number of factors was correctly specified, but not as a function of incorporating item discrimination parameters. Instead, the 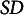 s of latent positions mostly decreased as more main parameters were employed in the model, indicating a reduction in CD.

**Figure 7:**
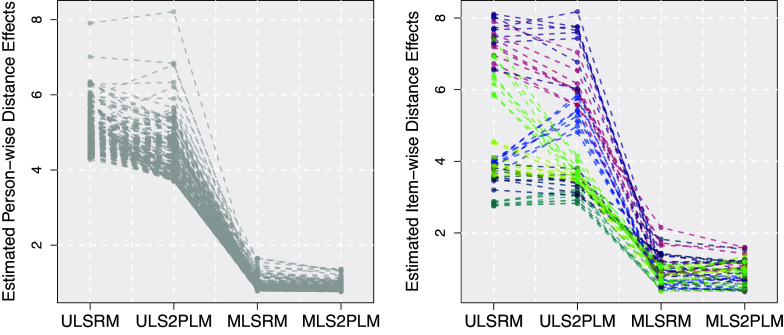
Reduction in the estimated distance effects 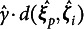 as a function of model complexity: The ADHD dataset. Left: Person-wise average distance effects. Right: Item-wise average distance effects.

Figure 7 provides a more thorough look at the decreases in the distance effects, person-wisely (left panel) and item-wisely (right panel). As in Figure 5, each dashed line corresponds to a single person or a single item. Replicating the findings from the IRDT dataset, persons and items showed generally decreasing extent of CD as a function of model complexity. The differences between unidimensional and multidimensional models were relatively small compared to those found in the IRDT dataset, which can be attributed to the fewer number of factors. However, rise and drop in the size of distance effects due to the factor dimensionality were consistently observed across most persons and items. Changes due to item discrimination were also consistent. Taken together, the four models applied to the ADHD dataset produced results similar to those observed in our first empirical examples, providing comparable implications on the relative nature of CD.

### 4.3 Qualitative differences in latent spaces across models

The primary reason we discussed and examined the relative nature of CD was its substantive implications in practical data analysis, particularly in studying unexplained person–item interactions and their implied individual differences, beyond the changes in the extent of CD. To illustrate these points, we revisit our empirical examples again. With the IRDT dataset, we show that a largely different item network can emerge on the latent space depending on which person and item parameters are incorporated in a model. With the ADHD dataset, we demonstrate that choices of main model parameters can lead to different configurations of latent positions, which in turn lead to different evaluations and diagnoses for respondents derived from CD.

#### 4.3.1 Changes in item networks due to main model parameters

**Figure 8:**
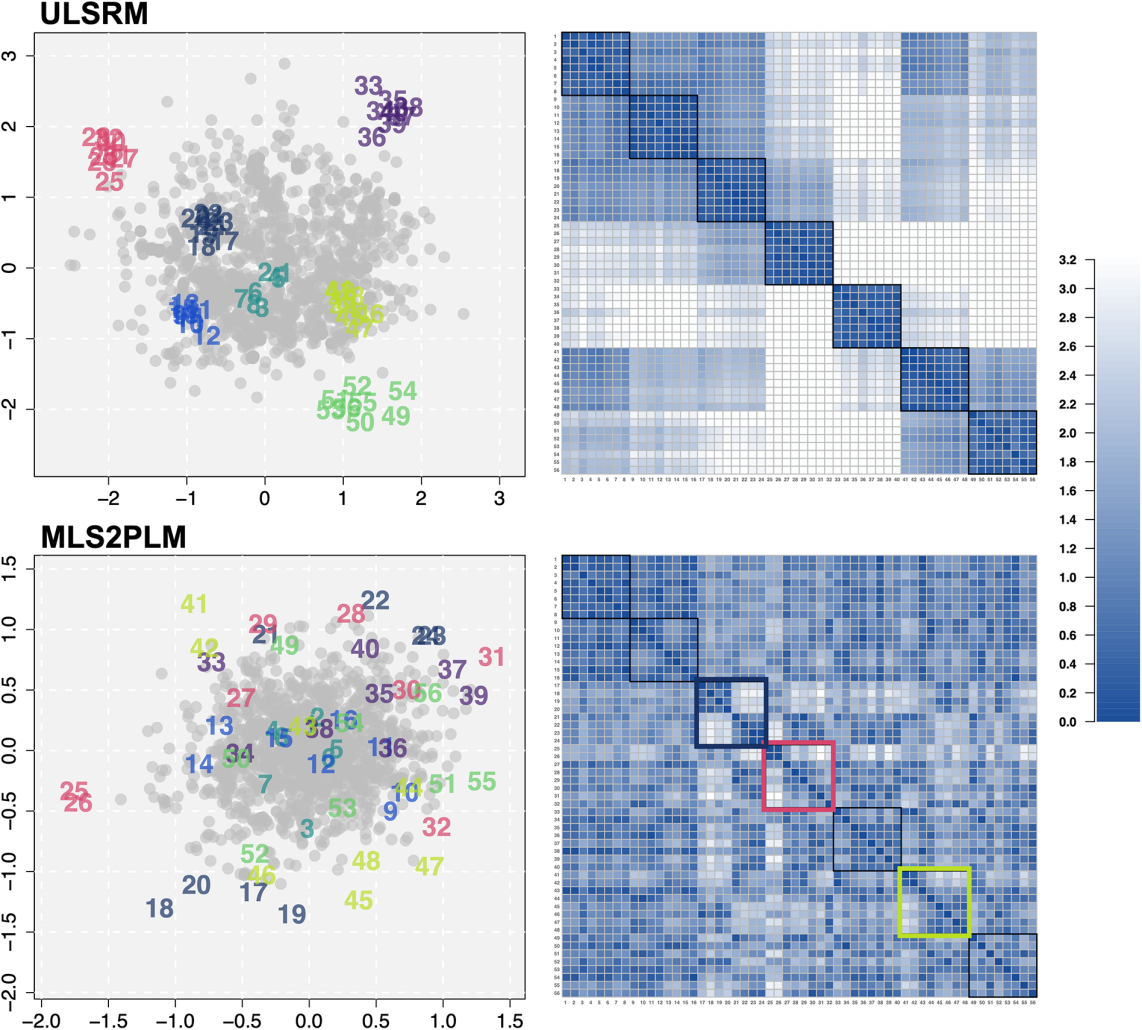
Estimated latent spaces for the IRDT dataset for the ULSRM and the MLS2PLM and their associated inter-item distance matrices.

To examine qualitative differences in the configurations of latent spaces and the patterns due to CD, Figure 8 presents two latent spaces of the IRDT dataset, obtained from ULSRM and MLS2PLM (left panels). These latent spaces were previously shown in Figure 4. However, unlike Figure 4, which uses the same *x*- and *y*-axis ranges for all estimated latent spaces to compare the extent of CD, Figure 8 removes this constraint, allowing each latent space to have its own axis ranges. As a result, the latent space of the MLS2PLM is zoomed in as its positions are more densely clustered compared to those in ULSRM. Also presented in Figure 8 are the inter-item distance matrices (right panels). For this matrix-like visualization of item networks, distances between items in the latent spaces were computed and color-coded according to the legend on the right side of Figure 8.

As described in Section 4.1, the ULSRM yielded the latent space (the top-left panel of Figure 8) in which item clusters emerged, each of which corresponds to the seven developmental stages considered in IRDT. This could be attributed to the unidimensional latent variable employed in the ULSRM which was not able to sufficiently capture variations due to factor differences. The inter-item matrix plot (the top-right panel of Figure 8) also confirms this pattern, showing a strong block-diagonal structure.

In contrast, the MLS2PLM yielded largely different configurations of latent positions. Most of all, there were seemingly no item clusters associated with the factor structure of IRDT, as judged by the item positions on the latent space (the bottom-left panel of Figure 8) and their inter-item distances (the bottom-right panel). This was because the variations across item clusters due to factor differences were effectively captured by the multidimensional latent variables. However, CD from the remaining variations still indicated substantial person–item interactions, as detected by the slab-and-spike prior with PIP 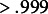 . This means that there were other sources of interactions not accounted for by the main model parameters of the MLS2PLM.

The latent space from the MLS2PLM allows more sophisticated analyses of unexplained interactions. Focusing on the inter-item interactions, a new interaction map reveals some heterogeneity between items measuring the same factor. The most clear pattern can be found from items 17–24 (color-coded as dark-blue), which exhibited two small groups, one on the bottom side (items 17–20) and the other on the top side of the latent space. The inter-item distance matrix also clearly showed this pattern, as highlighted by the thick dark-blue square. This represents that, even though these items were designed to assess the analogy at a specific developmental stage, they exhibited distinct item characteristics. A similar pattern can be found from items 41–48 (lime) as items 44–48 were located roughly around (0.5, 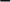 1.0), item 43 at the origin, and items 41 and 42 at the top side of the latent space. Also for items 25–32 (red), items 25 and 26 exhibited a unique association as they were located far left side of the latent space whereas the other items 27–32 were distributed roughly around (0.5, 0.5).

This investigation can be extended to person–person and person–item relationships to show substantive differences between different models due to the relativity in CD. For instance, as can be seen from the top-left panel of Figure 8, any single person would be judged to have similar distances to items 25–32 (red) by the ULSRM. In contrast, the bottom-left panel of the same figure shows that distances from the same person would vary to a large degree between items 25–26 and the other red items in the result by the MLS2PLM. In this way, different model structures and the relevant relativity in CD can produce largely distinct interpretations of CD and interactions between persons and items.

#### 4.3.2 Utilizing CD for personalized diagnoses: Dual importance of explained and unexplained data variations

We revisit the ADHD dataset to continue our illustration of the relative nature of CD and its consequences in substantive interpretations of person–item interactions. This time, we focus on possible differences in deriving personalized diagnoses and evaluations for different respondents. To this end, we selected two respondents, with ID numbers 94 and 120 (hereafter referred to as P94 and P120), for illustration.

**Figure 9:**
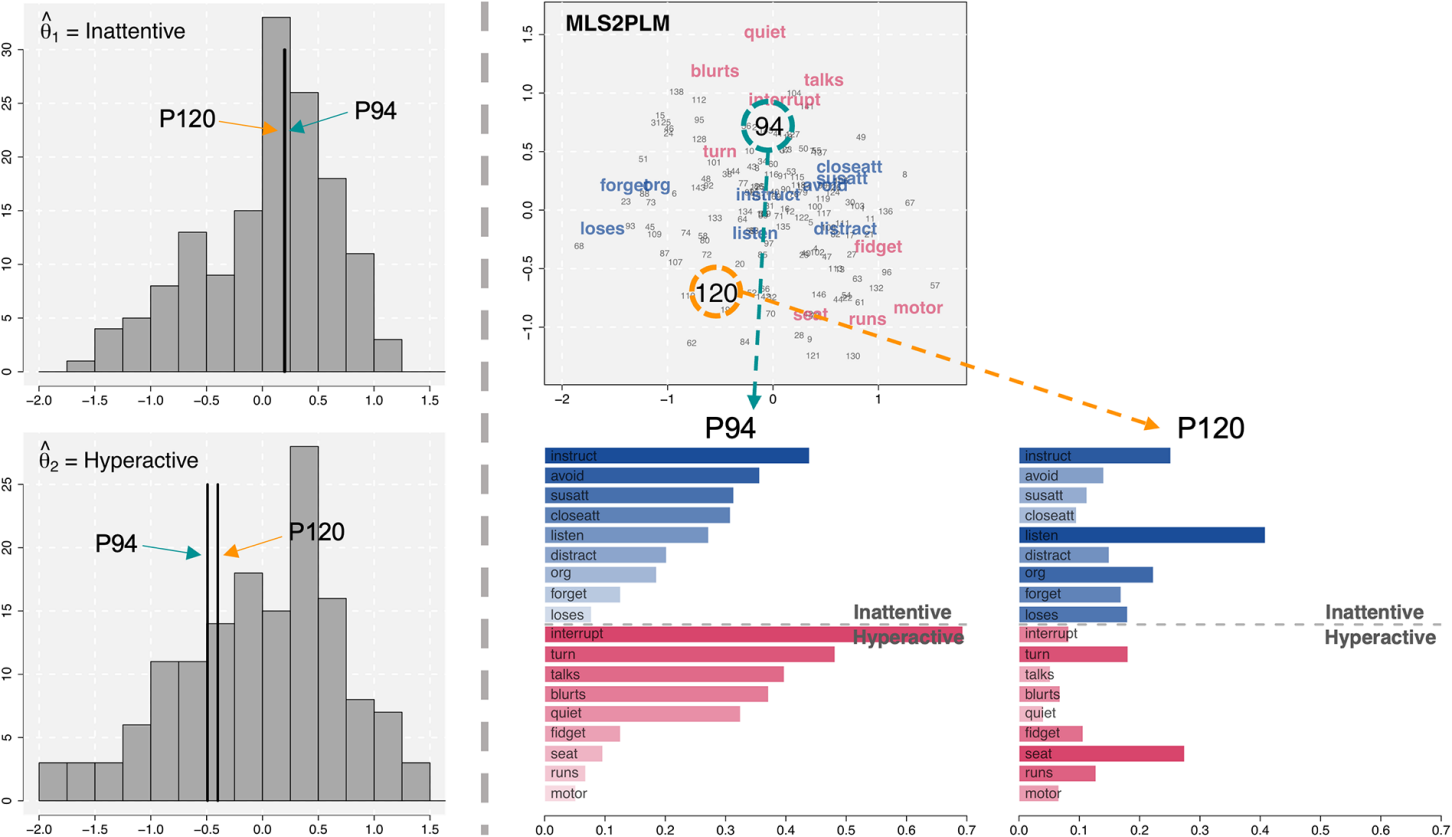
Factor score histograms, latent space, and individual symptom profiles from the MLS2PLM applied to the ADHD dataset.

The two histograms on the left side of Figure 9 display the distributions of the estimated latent traits 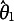 (inattentiveness) and 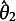 (hyperactiveness), representing the main systematic person effects. Within each histogram, two vertical lines indicate the locations of the latent scores for P94 and P120. As these lines show, the two selected respondents had nearly identical latent scores, meaning that their overall levels of inattentive and hyperactive symptoms were very similar.

The top-right panel of Figure 9 shows the latent space estimated from the MLS2PLM. This is the same as the one in Figure 6 but now estimated latent positions of P94 and P120 are highlighted with the enlarged ID numbers and the colored dashed circles surrounding them. Below the latent space, two bar charts for P94 (left) and P120 (right) are presented. These bar charts present the symptom similarity (i.e., the negative exponential transformation of a distance using Equation (4)) profiles of each respondent with different items. An individual bar corresponds to the similarity of the selected respondents to the item shown on the bar. The nine bluish bars on the top and the other nine reddish bars on the bottom correspond to inattentive and hyperactive symptoms, respectively. Also, darker colors indicate higher similarities (closer distances and stronger symptoms) while lighter colors represent lower similarities (farther distances and weaker symptoms). To facilitate comparison, items are ordered according to their similarities to P94 on the latent space estimated from the MLS2PLM.

Despite having nearly identical factor scores, the two respondents were located farther away from each other on the estimated latent space, particularly along the *y*-axis with 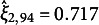 and 
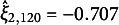
 . As a result, they had largely different profiles of person–item distances. For instance, P94 was closer to hyperactive symptoms on the top side of the latent space, such as *interrupt*, *turn*, and *talks* and accordingly showed higher similarities with them. In contrast, P120 was generally distant from the items, with the closest items being *listen* (inattentive) and *seat* (hyperactive). As higher similarities (i.e., smaller distances) are associated with higher probabilities of endorsing items, these differences between the two selected respondents suggest that their specific ADHD symptoms were notably distinct, despite their similar factor scores. This heterogeneity, uncaptured by estimated factor scores, can be further investigated with the latent space as illustrated and utilized to provide personalized diagnoses and feedback for different respondents.

**Figure 10:**
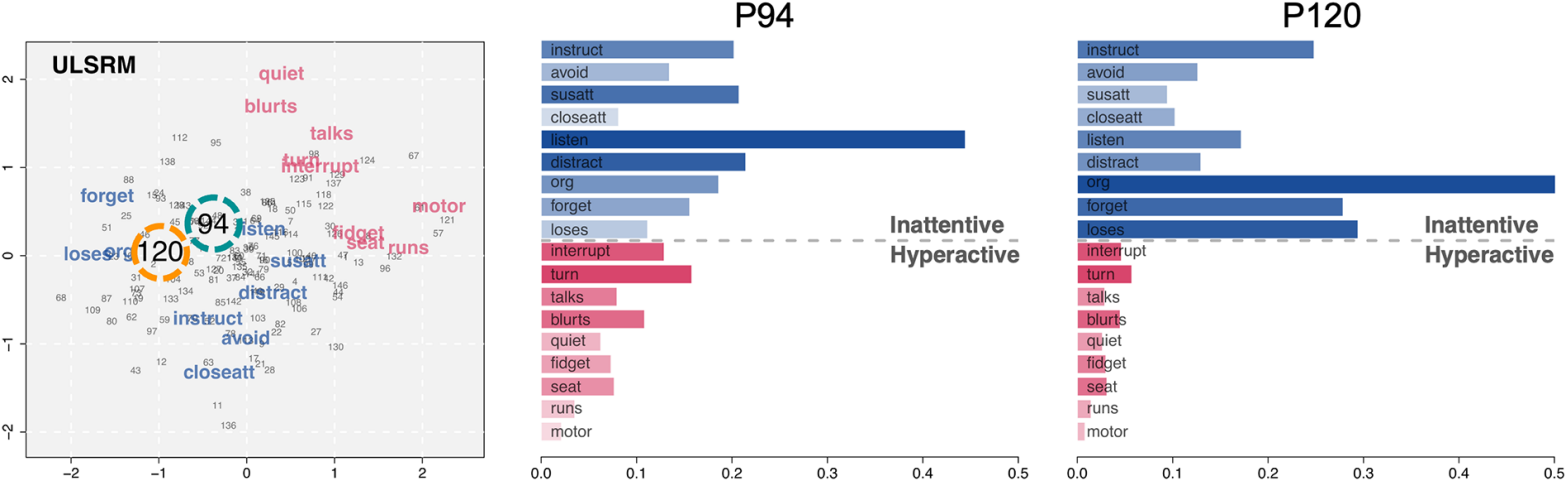
Individual symptom profiles from the ULSRM applied to the ADHD dataset.

The individual symptom profiles discussed thus far were based on the MLS2PLM. However, the relative nature of CD suggests that using a different model can considerably alter the results. To illustrate this, we examined the result from the ULSRM again. Figure 10 presents the relevant latent space as well as the individual symptom profiles of the two selected respondents, derived from the ULSRM. The single-factor scores of these two respondents were still very similar, with 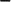 0.157 for P94 and 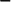 0.172 for P120.

Comparing the ULSRM results from those in Figure 9, it can be noticed that the individual profiles changed considerably. Three key differences emerged: (1) similarities were generally lower (i.e., larger distances) in the ULSRM results (see the range of the *x*-axis of the profiles), (2) latent positions of P94 and P120 were notably closer in the ULSRM results, leading to relatively similar profiles, and (3) both respondents have very low similarities to hyperactive symptoms, unlike those in the MLS2PLM results. Additionally, as can be seen by comparing changes in the distance profiles of P94 and P120, differences between models would also vary across respondents. Other respondents may show larger or smaller changes across models depending on their estimated latent positions. All these differences ultimately can lead to different diagnoses and evaluations based solely on the choice of model.

The individual symptom profiles underscore the value of studying CD, as they provide valuable insights into person–item interactions, individual differences, and personalized assessment of individuals (as well as items, although not shown). Taking this further, comparing the profiles from the two models (MLS2PLM and ULSRM) leads us back to the importance of considering the relative nature of CD. Models with varying complexities (due to the main model parameters) yield different levels of systematic explanations, producing different extents and configurations of CD, and consequently, different diagnoses, evaluations, and feedback. Also, some aspects of CD may reflect systematic variations that can be accounted for by suitable and interpretable main effect parameters (e.g., specifying factors with an appropriate dimension). Therefore, selecting a model with appropriate main effect parameters is crucial to simultaneously study the overall states of individuals (e.g., by factor scores) and person-wise specificities (e.g., by latent positions and distance effects).

## 5 Discussion

The proposed MLSIRMs extend the LSIRM with multidimensional factors and item discrimination just as the between-item multidimensional 2PLM does for the standard unidimensional Rasch model. The MLSIRMs analyze person and item effects underlying the data variations and quantify them as estimates of multidimensional factor scores, item discrimination parameters, and item difficulty parameters. Simultaneously, the models capture some of the residual data variations by means of latent space, representing them as scaled distances between persons and items mapped onto this space. This dual approach allows the models to account for person–item interactions unexplained by the main person and item effect parameters. The resulting information can be utilized to produce personalized diagnoses and evaluations for individuals. As described in our last empirical example section, this approach can uncover individual differences not revealed by factor scores.

While proposed as an extension of the LSIRM, the MLSIRMs also served as our framework to investigate and discuss the relative nature of CD. By imposing constraints on its main model parameters, the most complex MLS2PLM can be reduced to various simpler latent space models. This kind of constraint can limit the ability of a model to systematically explain data variations, leaving more residual variations unexplained. Consequently, a latent space of a simpler model would be given larger variations to capture. Conversely, when a more complex model with additional main parameters is used, less residual variations remain unaccounted for, leading to smaller estimates of distance effects. This pattern was demonstrated with both a simulation study and empirical examples.

In our illustrations with the IRDT and the ADHD datasets, the factor dimensionality turned out to yield relatively large differences in the estimated latent spaces and person–item interactions both in their sizes and configurations. Particularly for the IRDT dataset (Figure 4), the unidimensional models produced item clusters in the latent spaces corresponding to the misspecified dimensionality. In contrast, when the correct dimensionality was specified, the distribution of latent positions seemed more residual-like patterns. Looking deeper into the positions of items and their relative distances, however, it was possible to investigate differences in item-specific characteristics in their interactions with persons, even for the items measuring the same factors (Figure 8). The factor dimensionality was associated with relatively smaller differences in the latent spaces in the ADHD dataset (Figure 6) as there were only two factors considered. However, this dataset also revealed that more subtle (but statistically significant judged by the slab-and-spike prior) differences within the items measuring the same factors can be captured by the latent space when the correct dimensionality was specified. Generally, the item discrimination did not produce noticeable differences in the patterns/configurations of latent positions, but captured some of their variations, shrinking the positions toward the origin of the latent space and making them more densely gathered. However, the size of changes in CD due to factor dimensionality and item discrimination can vary across datasets, due to correlations of factors, variations in the range of item discriminations, etc. Whether the current findings are specific to our examples or can be generalized to other datasets (i.e., whether these are general properties of item discrimination parameters in terms of CD) should be further investigated.

Although we restricted the scope of our investigation to the dimensionality of factors and the item discrimination parameters in model extension as well as an examination of the relative property of CD, other model parameters used in psychometric models can be incorporated to further advance this line of research. For instance, random response, random guessing, and ability-based guessing can be other sources of CD (Bolsinova et al., 2017). If this is the case for a certain dataset, extending the current MLSIRMs with the pseudo-guessing parameters (also known as the lower asymptote parameters) in a three-parameter IRT model (Hambleton et al., 1978; Lord, 1980) and the upper asymptote in a four-parameter model (Barton & Lord, 1981; Hambleton & Swaminathan, 1985) could potentially partial out CD, explaining as data variations due to corresponding item effects. As a result, CD detected by the latent space can be reduced and accordingly, the configuration of the estimated interaction map can change. Similarly, if a test item of concern requires multiple problem-solving processes, a latent space model incorporating the concept of item complexity (Bolt & Liao, 2022; Samejima, 2000) could find a better balance between systematic and distance-based explanations of data variations rather than solely relying on what emerges on a latent space. However, this depends on whether at least some part of CD detected by the current model can be accounted for simply by main effects (not interactions) and whether the added parameters are suitable for capturing a subset of variations implied by CD.

The relative nature of CD implies that there could be potentially good alternative explanations when an interesting regularity is observed from CD. Variations attributed to CD in one model may, with appropriate modifications, turn out to be regular person or item effects. Most previous applications of the LSIRM and its variations have used the slab-and-spike prior to avoid greedily exploiting variations in item responses. However, even when this prior detects substantial CD, it is possible that some unexplained person–item interactions could be reinterpreted as systematic variations related to person and item characteristics. Certainly, a requisite for this possibility would be to find and incorporate appropriate main model parameters that offer reasonable and useful interpretations. Hence, it is crucial to balance model complexity due to person and item parameters in a model and examination of CD with relevant statistical integration. Although we focused on latent space modeling for this integration, the implication would apply to other statistical methods that attempt to move beyond the CI assumption and utilize CD to study unexplained person–item interactions and individual differences.

Future research can be dedicated not only to the issues of the relative nature of CD discussed above but also to some general topics of the latent space models in psychometrics. First, previous methods of examining CD and residual variations can be compared with the proposed approach. For instance, differential item functioning (DIF; Magis et al., 2010) and measurement (in)variance (Meredith, 1993) can be examined as interactions between items and groups of persons on the latent space. Unlike the traditional DIF and invariance testing methods, the latent space does not require to prespecify a group variable of interest. Also, latent positions can be linked to continuous variables so that invariance can be examined across degrees on such variables, in the sense of Molenaar (2021). As the latent space is employed to examine residual data variations and underlying unexplained person–item interactions, latent positions and their distances can be associated with traditional person fit and item fit indices (Emons et al., 2005; Reise, 1990; Sinharay, 2006). Furthermore, regarding the issue of the number of factors, patterns detected by the residual principal component analysis (Chou & Wang, 2010) can be compared with latent positions. Another important topic is the selection of *K*, the dimension of latent space. Most previous latent space modeling (both in network analysis and psychometrics) implemented 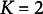 for easy visualization. However, to better account for CD and quantify unexplained person–item interactions, detecting the optimal dimension is necessary. Widely used Bayesian model selection criteria such as Deviance Information Criterion (Spiegelhalter et al., 2002), Watanabe-Akaike Information Criterion (Watanabe, 2010), Watanabe Bayesian Information Criterion (Watanabe, 2013), Leave-One-Out cross-validation (LOO; Vehtari et al., 2017) can be examined to see if they can accurately detect the optimal dimension of latent space. We hope that all these topics will be addressed soon, helping researchers easily utilize the proposed approach and better understand its relationship with traditional models.

## Supporting information

Kang and Jeon supplementary material Kang and Jeon supplementary material

## Footnotes

1 By “systematic”, we mainly refer to what can be explained by person and item effects when appropriate parameters are included in a model.

2 While we focus on parameters in psychometric models, it is also possible to add some person and item covariates (e.g., gender, length of item sentence, etc.) to account for person and item effects, as employed in explanatory item response theory (IRT) models (De Boeck & Wilson, 2004). Another exception to note is a person–item covariate. For example, if measured and incorporated in a model, response times  can capture some person–item interactions as they naturally vary across persons and items. However, these can also be considered systematic explanations, as they are explicitly included in a model.

3 Good performance of the slab-and-spike prior in the context of latent space modeling in psychometrics has been shown in Jeon et al. (2021) and Kang et al. (2023) by their simulation studies. We also conducted a similar simulation study, which confirms a comparable finding. The result can be found in our Supplementary Material (Section S4).

## Supplementary material

The supplementary material for this article can be found at https://doi.org/10.1017/psy.2025.5.
