# Supplementary material for: Multidimensional Latent Space Item Response Models: A Note on the Relativity of Conditional Dependence
Source: Psychometrika. 2025 Feb 26;90(2):799–826. doi: 10.1017/psy.2025.5 (PMC12483692; doi:10.1017/psy.2025.5)
Supplement: Kang and Jeon supplementary material [file S0033312325000055sup001.pdf]

MULTIDIMENSIONAL LATENT SPACE ITEM RESPONSE MODELS: A NOTE ON THE RELATIVITY OF CONDITIONAL INDEPENDENCE

S1. Stan Code to Fit a Multidimensional Latent Space Item Response Model: an MLS2PLM example

```
data {
  int<lower = 1> P;                  // number of persons
  int<lower = 1> I;                  // number of items
  int<lower = 1> N;                  // number of (non-NA) person-item pairs

  int<lower = 1, upper = P> pp[N];  // person index for the n-th obs
  int<lower = 1, upper = I> ii[N];  // item index for the n-th obs
  int<lower=0, upper=1> resp[N];    // response in the long format

  int<lower = 1> D;                  // number of factors
  int loading_idx[I, 2];             // index matrix for factor loadings
                                     // column 1: item numbers
                                     // column 2: factor numbers
  // Slab-and-spike hyperparameters
  real mu[2];        // two prior means for gamma: (-5, 0.5) in this article
  real kappa[2];     // two prior SDs for gamma: (1, 1) in this article
}
transformed data {
  vector[D] mu_theta = rep_vector(0.0, D);  // factor mean == fixed to 0

}
parameters {
  // main model parameters
  vector[D] theta[P];               // D-dim latent variables
  vector[I] log_a;                  // item discrimination
  vector[I] b;                      // item difficulty
  cholesky_factor_corr[D] Lcorr;    // for the factor cov
```

```
  // hyperparameters
  real mu_a;
  real<lower = 0> sig_a;
  real mu_b;
  real<lower = 0> sig_b;

  // coordinate in the latent space (K=2 for now)
  vector[P] xi1;
  vector[P] xi2;
  vector[I] zt1;
  vector[I] zt2;

  // tuning parameters for the latent space
  real log_gamma;                    // distance tuning parameter (log)
  real<lower = 0, upper = 1> pind;   // slab and spike probability
}
transformed parameters{
  corr_matrix[D] Phi;        // factor cov/corr mat (assuming standardized factors)
  matrix[I, D] a_mat;        // discrimination matrix
  vector[I] a = exp(log_a);
  real lambda;

  for(i in 1:I){
    for(d in 1:D){
      a_mat[i,d] = 0;
    }
  }
  for(i in 1:I){
    a_mat[loading_idx[i,1], loading_idx[i,2]] = a[i];
  }

  Phi = multiply_lower_tri_self_transpose(Lcorr);
  lambda = exp(log_lambda);
}
model {
```

```
  vector[2] lps;
  lps[1] = log(1-pind);
  lps[2] = log(pind);

  // prior distributions
  theta ~ multi_normal(mu_theta, Phi);
  log_a ~ normal(mu_a, sqrt(sig_a));
  b ~ normal(mu_b, sqrt(sig_b));
  Lcorr ~ lkj_corr_cholesky(1);
  mu_a ~ normal(0, 1);
  sig_a ~ cauchy(0, 5);
  mu_b ~ normal(0, 5);
  sig_b ~ cauchy(0, 5);

  pind ~ beta(1,1);
  xi1 ~ std_normal();
  xi2 ~ std_normal();
  zt1 ~ std_normal();
  zt2 ~ std_normal();

  // target distribution
  for(s in 1:2){
    lps[s] += normal_lpdf(log_lambda | mu[s], kappa[s]);
  }
  target += log_sum_exp(lps);
  for (n in 1:N) {
    resp[n] ~ bernoulli_logit(dot_product(a_mat[ii[n], 1:D],
        to_row_vector(theta[pp[n]])) +  b[ii[n]]
        - lambda * sqrt((xi1[pp[n]] - zt1[ii[n]])^2 + (xi2[pp[n]] - zt2[ii[n]])^2));
  }
}
```

S2. Additional Parameter Recovery Simulation Restuls

			$D=2$							$D=4$						
Measure	P	I_d	$\log(a_i)$	b_i	$\boldsymbol{\theta}_p$	$\boldsymbol{\Phi}$	$\boldsymbol{\xi}_p$	$\boldsymbol{\zeta}_i$	γ	$\log(a_i)$	b_i	$\boldsymbol{\theta}_p$	$\boldsymbol{\Phi}$	$\boldsymbol{\xi}_p$	$\boldsymbol{\zeta}_i$	γ
MSE	300	8	0.079	0.263	0.359	0.010	0.618	0.226	0.011	0.051	0.144	0.285	0.004	0.342	0.092	0.004
		16	0.045	0.189	0.255	0.001	0.353	0.101	0.004	0.039	0.227	0.216	0.003	0.216	0.106	0.002
	500	8	0.043	0.273	0.349	0.001	0.553	0.140	0.012	0.030	0.127	0.308	0.002	0.350	0.083	0.003
		16	0.028	0.111	0.247	0.001	0.360	0.066	0.002	0.024	0.103	0.209	0.001	0.187	0.054	0.001
	1000	8	0.024	0.116	0.356	0.001	0.508	0.056	0.008	0.020	0.082	0.309	0.001	0.345	0.044	0.003
		16	0.017	0.088	0.230	0.000	0.352	0.040	0.002	0.015	0.069	0.202	0.002	0.186	0.030	0.001
Bias	300	8	0.145	0.336	0.354	0.079	0.498	0.205	0.028	0.117	0.169	0.300	0.034	0.278	0.111	0.036
		16	0.088	0.256	0.280	0.020	0.282	0.119	0.005	0.091	0.216	0.239	0.037	0.184	0.084	0.013
	500	8	0.084	0.315	0.355	0.011	0.417	0.163	0.010	0.062	0.167	0.327	0.020	0.279	0.096	0.011
		16	0.058	0.117	0.273	0.009	0.273	0.074	0.022	0.056	0.140	0.227	0.018	0.154	0.081	0.007
	1000	8	0.041	0.177	0.355	0.021	0.388	0.075	0.040	0.047	0.137	0.318	0.019	0.274	0.084	0.035
		16	0.045	0.121	0.248	0.002	0.284	0.056	0.018	0.056	0.098	0.212	0.035	0.153	0.044	0.006
SE	300	8	0.222	0.343	0.374	0.062	0.428	0.320	0.103	0.185	0.296	0.362	0.051	0.430	0.254	0.050
		16	0.175	0.305	0.339	0.027	0.435	0.260	0.066	0.159	0.311	0.340	0.033	0.369	0.239	0.048
	500	8	0.179	0.335	0.357	0.034	0.458	0.271	0.108	0.150	0.267	0.356	0.035	0.440	0.225	0.054
		16	0.140	0.267	0.336	0.022	0.441	0.227	0.043	0.136	0.261	0.335	0.024	0.356	0.199	0.032
	1000	8	0.144	0.277	0.371	0.029	0.463	0.212	0.079	0.124	0.222	0.362	0.028	0.433	0.170	0.045
		16	0.112	0.223	0.346	0.016	0.440	0.163	0.044	0.099	0.211	0.340	0.021	0.356	0.148	0.029

Table S1: Parameter recovery results of the proposed model (when $\phi_{jl} = 0.00$)

			$D=2$							$D=4$						
Measure	P	I_d	$\log(a_i)$	b_i	$\boldsymbol{\theta}_p$	$\boldsymbol{\Phi}$	$\boldsymbol{\xi}_p$	$\boldsymbol{\zeta}_i$	γ	$\log(a_i)$	b_i	$\boldsymbol{\theta}_p$	$\boldsymbol{\Phi}$	$\boldsymbol{\xi}_p$	$\boldsymbol{\zeta}_i$	γ
MSE	300	8	0.076	0.272	0.403	0.014	0.630	0.245	0.013	0.050	0.160	0.356	0.007	0.343	0.101	0.004
		16	0.048	0.182	0.270	0.003	0.341	0.100	0.003	0.042	0.201	0.252	0.007	0.209	0.103	0.001
	500	8	0.046	0.247	0.375	0.003	0.544	0.130	0.011	0.035	0.134	0.385	0.003	0.345	0.086	0.002
		16	0.030	0.107	0.263	0.002	0.349	0.066	0.002	0.022	0.099	0.235	0.002	0.183	0.051	0.002
	1000	8	0.023	0.105	0.390	0.004	0.502	0.053	0.006	0.020	0.081	0.373	0.002	0.334	0.040	0.002
		16	0.017	0.086	0.244	0.001	0.348	0.041	0.002	0.013	0.063	0.232	0.005	0.179	0.028	0.000
Bias	300	8	0.124	0.308	0.364	0.091	0.509	0.223	0.017	0.102	0.189	0.319	0.047	0.283	0.135	0.007
		16	0.103	0.243	0.274	0.032	0.271	0.111	0.010	0.092	0.200	0.234	0.067	0.174	0.096	0.005
	500	8	0.086	0.311	0.344	0.008	0.409	0.154	0.014	0.067	0.171	0.346	0.023	0.277	0.116	0.019
		16	0.063	0.102	0.262	0.020	0.266	0.070	0.012	0.047	0.125	0.215	0.019	0.148	0.072	0.002
	1000	8	0.036	0.154	0.357	0.048	0.388	0.065	0.024	0.045	0.134	0.325	0.024	0.267	0.076	0.021
		16	0.048	0.121	0.240	0.006	0.282	0.063	0.012	0.037	0.090	0.203	0.060	0.149	0.043	0.004
SE	300	8	0.220	0.358	0.407	0.072	0.431	0.327	0.112	0.187	0.294	0.417	0.063	0.430	0.252	0.063
		16	0.169	0.307	0.362	0.045	0.435	0.262	0.055	0.160	0.305	0.382	0.045	0.371	0.242	0.036
	500	8	0.186	0.311	0.398	0.049	0.462	0.266	0.102	0.163	0.266	0.417	0.050	0.439	0.221	0.043
		16	0.142	0.274	0.367	0.033	0.443	0.230	0.048	0.133	0.256	0.379	0.033	0.357	0.196	0.040
	1000	8	0.140	0.275	0.404	0.042	0.463	0.212	0.074	0.124	0.227	0.427	0.035	0.432	0.165	0.040
		16	0.110	0.219	0.374	0.022	0.442	0.164	0.040	0.103	0.202	0.381	0.024	0.352	0.145	0.021

Table S2: Parameter recovery results of the proposed Model (when $\phi_{jl} = 0.75$)

S3. Model Predictive Accuracy Under Conditional Dependence

Continuing from Section *Impact of Ignoring Conditional Dependence* in the main manuscript, we further investigate how CD influences our estimations and inferences but now focusing on model predictions. To this end, we performed posterior predictive checking (PPC) of the MLS2PLM and the MIRM with the synthetic datasets for the four conditions, used to generate Figure 1 in the main manuscript. For each of the models, we randomly selected 1000 posterior samples of the model parameters from each repetition of the simulation conditions. Then we generated a $(P \times I)$ item response matrix from each sample, which resulted in $(1000 \times P \times I)$ posterior predictive samples per repetition. These samples were summarized and contrasted to the data-based item-wise and person-wise response proportions.

First, we examined if the MLS2PLM can outperform the MIRM across repetitions and simulation conditions. For that purpose, we computed both predictive item-wise and person-wise proportions for each of the predictive samples, yielding a $(1000 \times I)$ item-wise proportion matrix and a $(1000 \times P)$ person-wise proportion matrix. Then, absolute deviations from data-based pro-

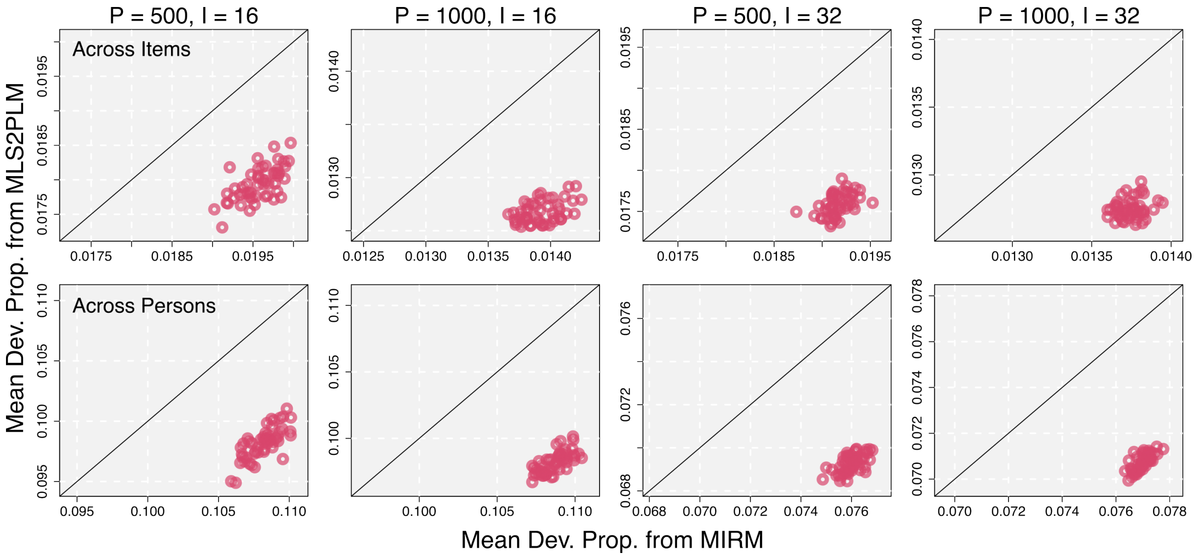

Figure S1: Scatterplots of absolute deviations in response proportion. Upper panels: Deviations in item-wise proportion were computed and averaged across items, for each of the repetitions in a simulation condition (denoted on top of the upper panels). Lower panels: Deviations in person-wise proportion were computed and averaged across persons, for each of the repetitions in a simulation condition.

portions were computed and averaged across 1000 predictive samples, resulting in I item-wise deviations and P person-wise deviations per repetition. Finally, item-wise absolute deviations were averaged across items and person-wise averaged across persons. Because the number of repetitions was 50, this produced 50 summary statistics of predictive accuracy for item-wise response proportions and another 50 statistics for person-wise proportions.

The results are presented in Figure S1. Mean deviations in proportion predicted by the MIRM are plotted on the x-axis against those from the MLS2PLM on the y-axis. The upper and the lower panels present the results for item-wise and person-wise response proportions, respectively. Also, the four simulation conditions are denoted on top of the upper panels. The diagonal line in each panel indicates where the values on the x-axis are equal to those on the y-axis.

In all panels, the red dots are located on the far bottom-right side of the diagonal line. This indicates that the mean deviations in proportion are larger in the MIRM. Thus, the results strongly support that the proposed MLS2PLM can effectively reduce the deviations between observations and model predictions.

Regarding the results in Figure S1. It can be questioned how much the improvement by the

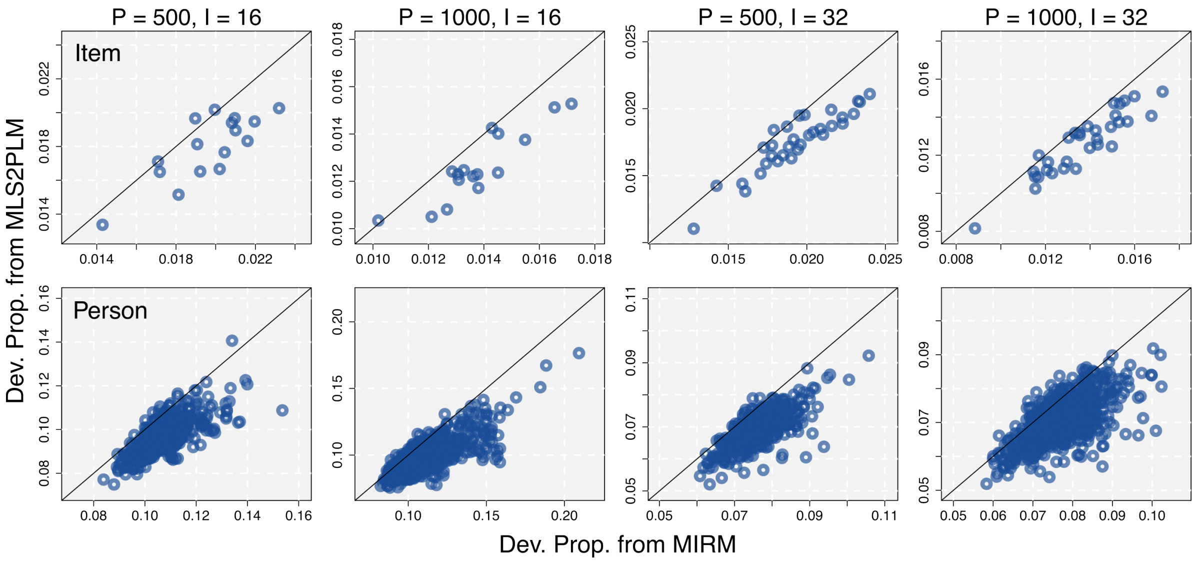

Figure S2: Scatterplots of absolute deviations in item-wise and person-wise response proportions in a selected replication of the simulation conditions. Top panels: Deviations in item-wise proportions in the first replication of the simulation conditions (denoted on top of the columns). Bottom panels: Deviations in person-wise proportions in the first replications of the simulation conditions.

proposed model depends on items and persons. To further look into the results, we chose the first synthetic datasets per condition and examined absolute deviations of item-wise and person-wise predicted proportions from the corresponding observations, averaged across posterior predictive samples.

The results are presented in Figure S2, which has a very similar configuration as Figure S1. The only difference is that now (blue) dots indicate items (upper panels) and persons (lower panels) and these are from the first repetition of the simulation conditions. The scatterplots show that most of the dots are on the bottom-right side of the diagonal lines, indicating that the MLS2PLM can yield better predictions for most items and persons.

Finally, we examined if a similar result can be obtained from real-world data. To this end, we performed the same PPC to the model estimation results of the ADHD dataset used in the main manuscript. The results are presented in Figure S3. The left panel compares the averaged absolute deviation in item-wise response proportions between the two models (the MIRM on the x-axis and the MLS2PLM on the y-axis) and the right panel compares those for the person-wise proportions. The finding is consistent, as most deviations are on the bottom-right side of the diagonal lines, indicating that the proposed model outperformed the traditional MIRM. The difference can be attributed to the CD underlying data, as the only difference between the models is that the MLS2PLM can capture this CD with the latent space.

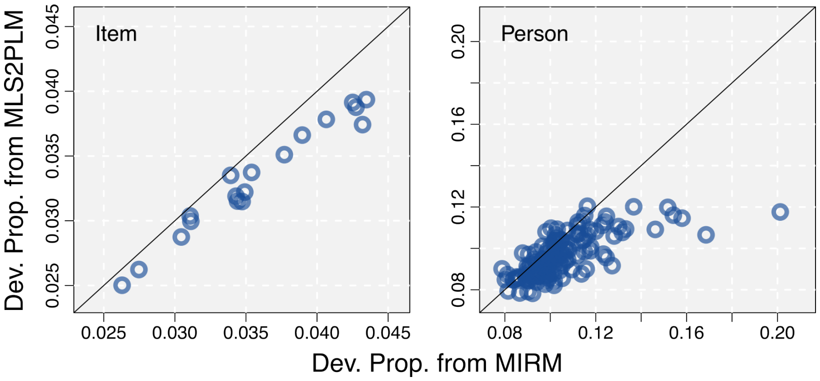

Figure S3: Scatterplots of absolute deviations in item-wise and person-wise response proportions in the ADHD dataset. Left panel: Deviations in item-wise proportions. Right panels: Deviations in person-wise proportions.

S4. Performance of Model Selection by Slab-and-Spike Prior

In this section, we present the results of the simulation study examining the accuracy of the slab-and-spike prior implemented in the proposed model. That is, we aim to see if the model selection feature of the model can correctly remove the latent space if data do not imply CD and if it can correctly detect CD when there is substantial CD underlying data. For this purpose, we generated data from the MLS2PLM with $\gamma = 0.0$ (no CD condition) and $\gamma = 1.5$ (substantial CD condition. Note that, for $\gamma = 0.0$, the data-generating model is just the same as the traditional MIRM. We chose $P = 500$ and $I = 16$ (also used in all the simulation studies in the main manuscript and Section S3 in this supplementary material). For each condition, we generated 50 synthetic datasets and fitted the MLS2PLM. Then, we computed the posterior inclusion probabilities $(P(\delta = 1 \mid \boldsymbol{Y}))$ for each repetition, using Equation 5 in the main manuscript.

The results are presented in Figure S4. The left and the right panels present histograms of PIPs obtained under the conditions with $\gamma = 0.0$ and $\gamma = 1.5$, respectively. Judging from the distribution of PIPs, it can be concluded that the MLS2PLM can accurately select the spike part when there is no clear evidence of CD in the data and also can correctly choose to employ a latent space when substantial CD can be detected.

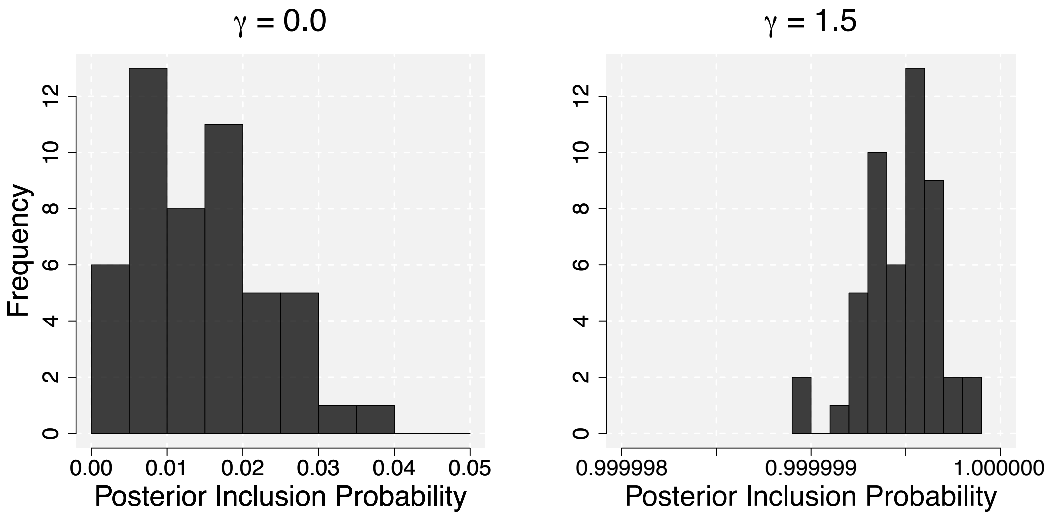

Figure S4: Histograms of posterior inclusion probabilities $P(\delta = 1 \mid \boldsymbol{Y})$: when the data-generating value of γ is 0 (left) and 1.5 (right)

S5. Convergence of Bayesian Chains

Convergence of Bayesian chains was first assessed with potential scale reduction statistics ($\hat{R}$; Gelman, 1996; Gelman, Carlin, Stern, Dunson, & A. Vehtari, 2013). For our empirical examples, histograms of $\hat{R}$ obtained from the MLS2PLM are presented in Figure S5, which shows that all $\hat{R}$ values are below the typical cutoff value of 1.1. Figures S6 and S7 present trace plots of Bayesian samples for several randomly selected parameters, which also support reasonable convergence of the chains. Convergence of the other models was also examined analogously, not exhibiting a bad convergence issue in the final estimation results.

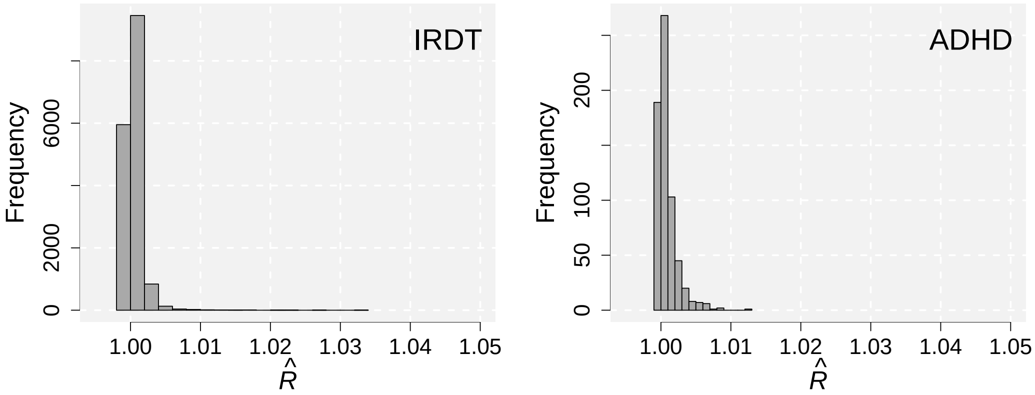

Figure S5: Histograms of potential scale reduction statistics ($\hat{R}$)

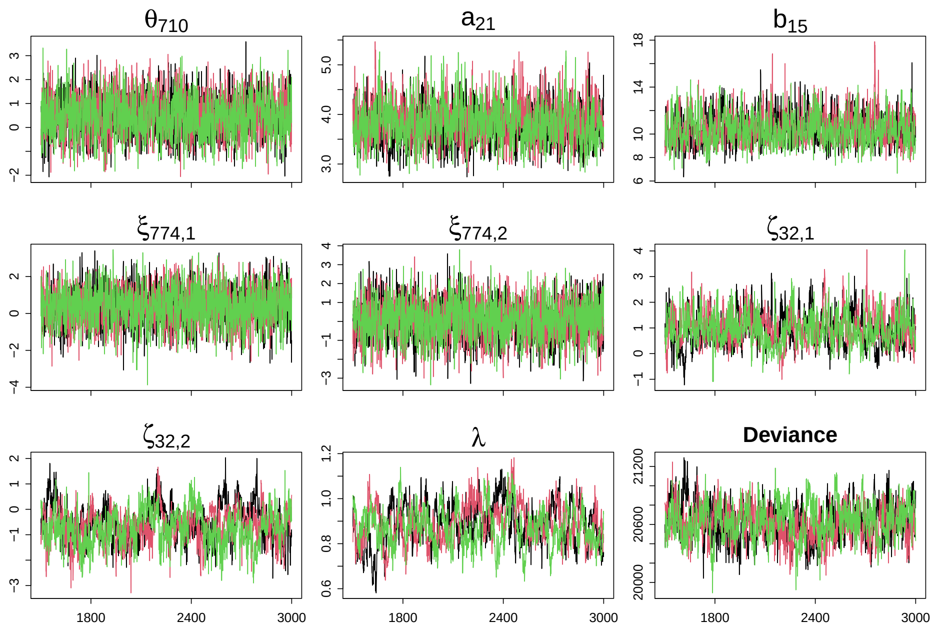

Figure S6: Trace plots of randomly selected parameters (IRDT dataset).

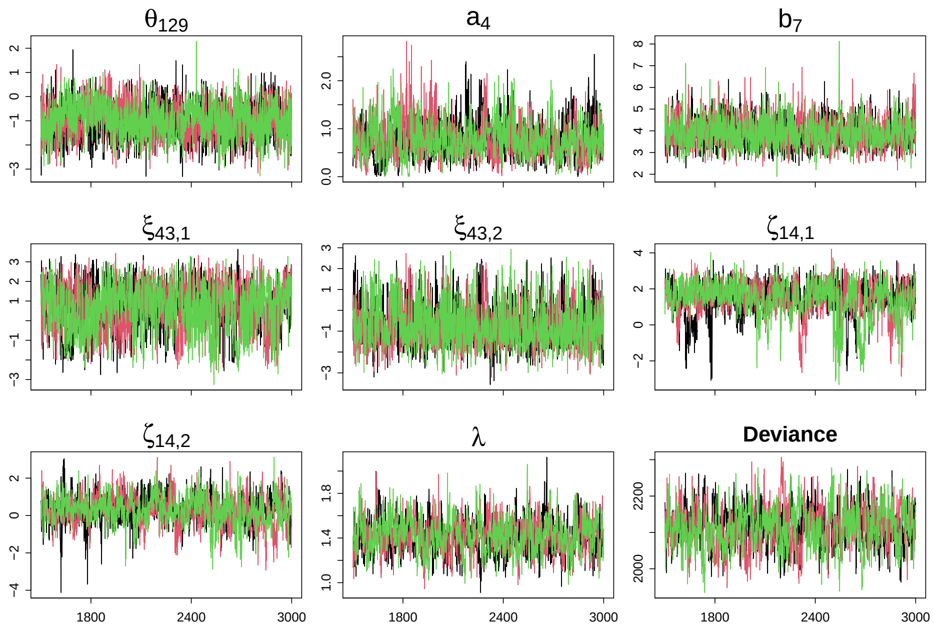

Figure S7: Trace plots of randomly selected parameters (ADHD dataset).

S6. Miscellaneous

S6.1. Symptom Items in the ADHD Dataset

Table S3: Symptom Items in the ADHD dataset

Item	Symptom
**Inattentive**	
closeatt	Often fails to give close attention to details or makes careless mistakes in schoolwork, work, or other activities (fails close attention)
susatt	Often has difficulty sustaining attention in tasks or play activities (difficulty sustaining attention)
listen	Often does not seem to listen when spoken to directly (does not listen)
instruct	Often does not follow through on instructions and fails to finish schoolwork, chores, or duties in the workplace (does not follow instructions)
org	Often has difficulty organizing tasks and activities (difficulty organizing)
avoid	Often avoids, dislikes, or is reluctant to engage in tasks that require sustained mental effort (avoids mental effort)
loses	Often loses things necessary for tasks or activities (loses things)
distract	Is often easily distracted by extraneous stimuli (easily distracted)
forget	Is often forgetful in daily activities (forgetful)
**Hyperactive/Impulsive**	
fidget	Often fidgets with hands or feet or squirms in seat (fidgets)
seat	Often leaves seat in classroom or in other situations in which remaining seated is expected (leaves seat)
runs	Often runs about or climbs excessively in situations in which it is inappropriate (runs excessively)
quiet	Often has difficulty playing or engaging in leisure activities quietly (difficulty playing quietly)
motor	Is often "on the go" or often acts as if "driven by a motor" (driven by motor)
talks	Often talks excessively (talks)
blurts	Often blurts out answers before questions have been completed (blurts)
turn	Often has difficulty awaiting turn (awaiting turn)
interrupt	Often interrupts or intrudes on others (interrupts)

S6.2. Interitem Distance Analysis

One way of quantitatively exploring estimated latent spaces is to use distances between persons, between items, and/or between persons and items. In this section, we present matrix-like visualizations of inter-item distances of the latent spaces from our empirical examples. We chose inter-item distances simply because the number of items is typically much smaller than the number of persons, and so they can provide clearer visualization results. However, one can produce similar visualization results of inter-person or person-item distances for some selected persons and items. Alternatively, one can directly use the computed numeric values of distance effects to make inferences regarding person-to-person, item-to-item, and person-to-item interactions.

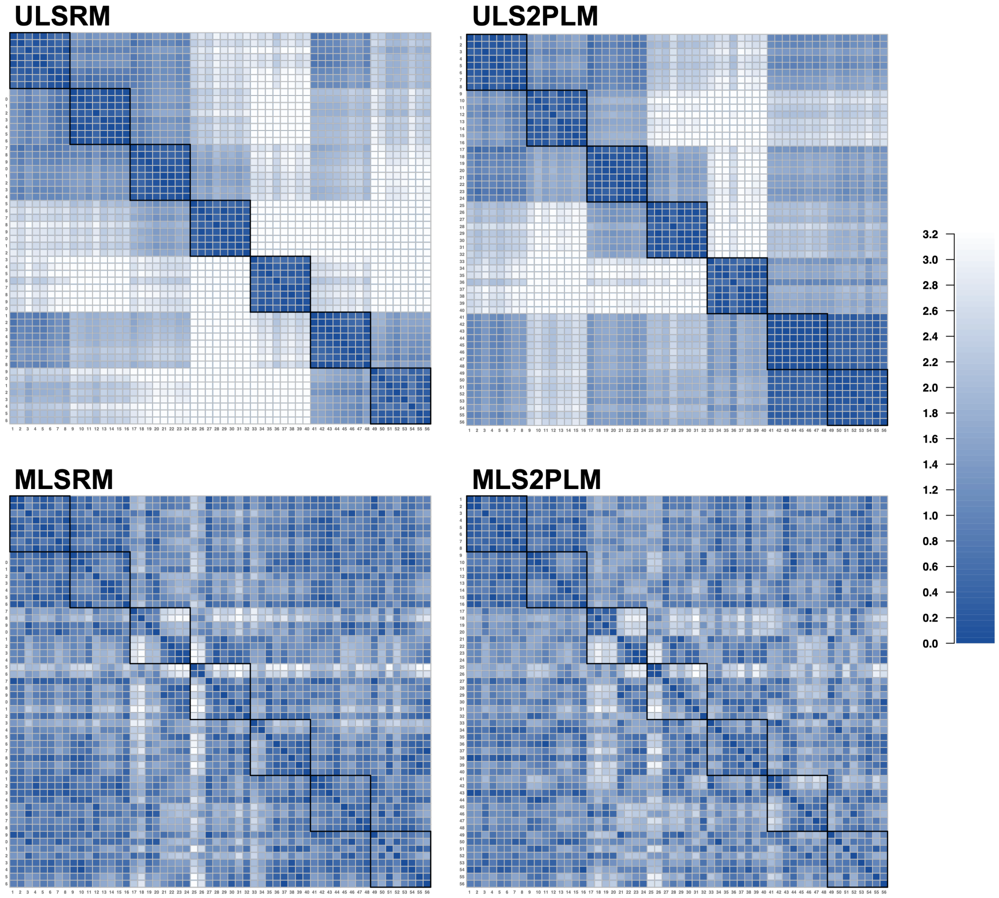

Figure S8: Inter-item distance matrices for the IRDT dataset

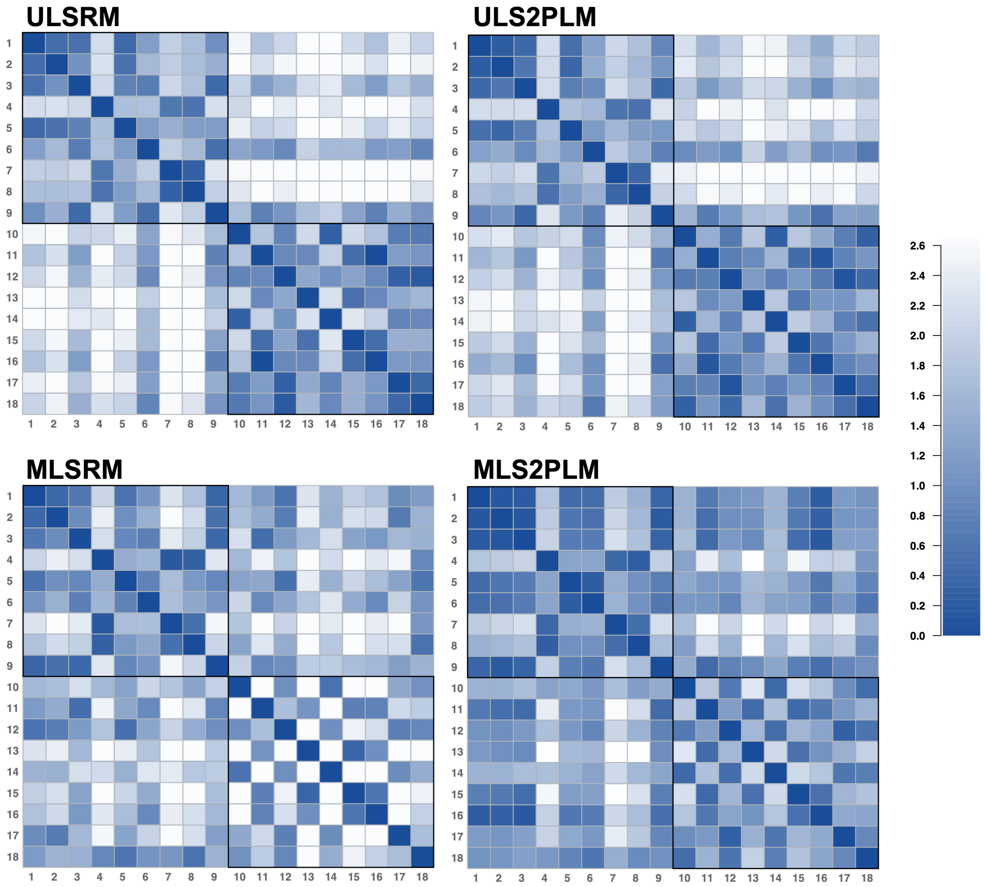

Figure S9: Inter-item distance matrices for the ADHD dataset

References

Gelman, A. (1996). Inference and monitoring convergence. In W. R. Gilks, S. Richardson, & D. J. Spiegelhalter (Eds.), *Markov chain monte carlo in practice* (p. 131-143). CRC Press.

Gelman, A., Carlin, J. B., Stern, H. S., Dunson, D. B., & A. Vehtari, D. B. R. (2013). *Bayesian data analysis* (3rd ed.). CRC Press.
